# Supplementary material for: Effects of restricting social media usage on wellbeing and performance: A randomized control trial among students
Source: PLoS One. 2022 Aug 24;17(8):e0272416. doi: 10.1371/journal.pone.0272416 (PMC9401146; doi:10.1371/journal.pone.0272416)
Supplement: S1 Fig — (DOCX) [file pone.0272416.s001.docx]

**Figure S1: All digital activities and social media usage over time (all users)**

black = treatment, white = control group
solid vertical lines: start of a new teaching block
dashed vertical lines: start of the exam period
