# Supplementary material for: Effects of restricting social media usage on wellbeing and performance: A randomized control trial among students
Source: PLoS One. 2022 Aug 24;17(8):e0272416. doi: 10.1371/journal.pone.0272416 (PMC9401146; doi:10.1371/journal.pone.0272416)
Supplement: S2 Fig — a Tracked digital activities over time (all users). b. Tracked digital activities over time (all users). (DOCX) [file pone.0272416.s002.docx]

**Figure S2.1: Tracked digital activities over time (all users)**

black = treatment, white = control group
solid vertical lines: start of a new teaching block
dashed vertical lines: start of the exam period

**Figure S2.2: Tracked digital activities over time (all users)**

black = treatment, white = control group
solid vertical lines: start of a new teaching block
dashed vertical lines: start of the exam period
