# Supplementary material for: Effects of restricting social media usage on wellbeing and performance: A randomized control trial among students
Source: PLoS One. 2022 Aug 24;17(8):e0272416. doi: 10.1371/journal.pone.0272416 (PMC9401146; doi:10.1371/journal.pone.0272416)
Supplement: S1 Table — (DOCX) [file pone.0272416.s003.docx]

**Table S1: Significance of differences between treatment and control group in block 2 vs. block 1 (difference-in-differences)**

| Category | p-value all users | p-value Android users |
| --- | --- | --- |
| Social media | **<0.001** | **0.013** |
| General Reference & Learning | 0.811 | 0.491 |
| Instant Message | 0.123 | **0.008** |
| Browsers | 0.628 | 0.257 |
| Video | 0.513 | 0.715 |
| Writing | 0.532 | 0.304 |
| Search | 0.745 | 0.494 |
| Email | 0.352 | 0.104 |
| News & Opinion | 0.864 | 0.543 |
| General Entertainment | 0.679 | 0.506 |
| Games | 0.411 | 0.662 |
| Presentation | 0.857 | 0.803 |
| General Shopping | 0.833 | 0.611 |
| Music | 0.099 | **0.027** |

All variables measured on a log scale.
