# Supplementary material for: Effects of restricting social media usage on wellbeing and performance: A randomized control trial among students
Source: PLoS One. 2022 Aug 24;17(8):e0272416. doi: 10.1371/journal.pone.0272416 (PMC9401146; doi:10.1371/journal.pone.0272416)
Supplement: S2 Table — (DOCX) [file pone.0272416.s004.docx]

**Table S2: Summary statistics for well-being measures**

| Measure | Full sample | | Treatment group | | Control group | |
| --- | --- | --- | --- | --- | --- | --- |
|  | Mean | SD | Mean | SD | Mean | SD |
| SWLS survey 1 | 25.0 | 5.5 | 24.3 | 5.9 | 25.7 | 5.1 |
| SWLS survey 2 | 25.0 | 5.4 | 24.4 | 5.4 | 25.6 | 5.3 |
| SWLS survey 3 | 25.1 | 5.3 | 24.4 | 5.3 | 25.7 | 5.3 |
| SWEMWBS survey 1 | 22.8 | 3.0 | 23.0 | 2.8 | 22.5 | 3.1 |
| SWEMWBS survey 2 | 22.5 | 2.7 | 22.4 | 2.6 | 22.5 | 2.9 |
| SWEMWBS survey 3 | 22.4 | 3.3 | 22.4 | 3.6 | 22.5 | 3.1 |
