# Supplementary material for: Effects of restricting social media usage on wellbeing and performance: A randomized control trial among students
Source: PLoS One. 2022 Aug 24;17(8):e0272416. doi: 10.1371/journal.pone.0272416 (PMC9401146; doi:10.1371/journal.pone.0272416)
Supplement: Attachment — Submitted filename: PONE-D-21-20386-review.pdf [file pone.0272416.s005.pdf]

This paper describes a random assignment experiment comparing the subjective well-being (satisfaction with life and mood) and grades of university students who were asked to reduce their social media use (Facebook, Instagram, Snapchat) to less than 10 minutes per day with a control group who were not asked to make social media adjustments. Results show no significant differences between the reduction group and control group on these outcomes. (Because I am not familiar with work that on social media use and educational outcomes, I will ignore it in this review.)

As the authors note, prior research on the impact of social media use on subjective well-being has primarily involved cross-sectional surveys, correlating self-reports of social media use with self-reports of well-being. Moreover, again as the authors note, results from the correlational research have been mixed, although the most recent meta-analysis reviewing 94 studies (Liu et al., 2019) suggests that on average social media use is negatively associated with subjective well-being. However, the association is substantially dependent on type of use (e.g., r=.14 for social interaction versus -.14 for content consumption). Thus, a random assignment experiment is an excellent addition to this body of work.

The authors introduce their research with the claim for novelty. "To the best of our knowledge, this study is the first that tracks all of these components of well-being and over a long period of time." This claim is overstated. I am familiar with five experiments, in which participants were randomly assigned to reduce their social media use for one to four weeks (Allcott et al., 2020; Brailovskaia et al., 2020; Hanley et al., 2019; Mosquera et al., 2019; Vanman et al., 2018). The current research improves on prior experiments by upping the ante to nine weeks. Although some of the prior experiments used small student samples like the current study, others used much larger and more diverse samples. All of them show that social media restrictions led to better subjective well-being. Of these, the current authors cite only the Allcott paper, but criticize it because it only reduced social media use for a four-week period.

I have some substantive methodological concerns about the paper involving the conceptualization of the intervention, statistical methods, and the power of the intervention. Given the prior literature, which suggests that the impact of social media use depends upon the functions for which it is used and not just not the total amount of use, I am disappointed that the current research did not target more precisely the type of social media use to restrict. For example, a 2 X 2 factorial experiment (reduce social interaction vs control (reduce content consumption vs control) would have been more informative than the current experiment, which restricted overall use, and could have been done even with a relatively small sample size of 130 completed participants.

I also disappointed on the way that the research defined social media use for participants. The research considered Facebook messenger (a popular application on Facebook), Instagram and Snapchat, all of which are used for one-on-one or small group communication, as social media applications while WhatsApp, another messaging application, is not classified as social media.

According to the methods description on page 13 and Tables 4 and 5 in the manuscript, the authors have some data on what activities participants used across the platforms. I think the authors could more strongly advance the prior literature about the differential importance of types of use and still take advantage of their random assignment experiment by using instrumental variable regression or mediation models to analyze the data. For example, the authors could use the experiment to dig more deeply into the conclusion form Liu et al's meta-analysis that social interaction and content consumption have different effects on well-being. An instrumental variable regression analysis could examine the extent to which the experimental manipulation changed participants' type of use (e.g.,

instant messaging and email as proxies for use for social interaction vs general news, general shopping, and video as proxies for content consumption) and how the changes in in use for social interaction and content consumption use caused by the manipulation predict well-being and grades.

I have two concerns about the power of the experiment. First, the manipulation check indicates that the experimental manipulation did not have a major impact on the amount of time participants spent on social media, with participants in the experimental condition reducing time spent on the three predefined social media applications by 13 minutes versus 3.7 minutes for participants in the control condition. Given the substitution behavior the authors document, in which participants in experimental condition started using WhatsApp more, the experimental manipulation did not reduce overall social media use. Instead, it caused shifts in the types of social media applications used, if one considers Facebook, Instagram, Snapchat, and WhatsApp all as social media platforms. This shift in behavior is another reason I recommend that the authors consider an instrumental variable regression model to test their hypotheses.

Second, both the correlational and experimental prior literature suggests that the relationship between social media use and well-being is likely to be small, with the average absolute correlation being -.10 in the Liu meta-analysis of correlational studies and a .09 standard deviation (STD) effect size in the Allcott et al experiment. Given the likely small effect size, an experiment with 130 participants is probably underpowered to detect this effect. The authors' discussion of their power analysis indicates that their study could detect a .6 STD effect on the SWLS Life Satisfaction scale, substantially larger than the effect size one might have anticipated from the prior literature. While the authors acknowledge this limitation, they still interpret their failure to find a significant effect of their restriction intervention much more seriously than it deserves. As their paper states, "Contrary to findings from previous correlational studies, we do not find any impact of social media usage on wellbeing and academic success. … While we found null results estimating the causal impact of social media usage on well-being and academic performance, and not all null results matter, we believe that null results are interesting and important in this context. The media has hyped correlational studies showing a negative association between social media usage and well-being and it is important to balance this narrative through causal evidence." Indeed, even though the experiment found no significant effects of their intervention on either satisfaction with life or mental well-being, the treatment X Block2 effects are both negative and consistent with the prior literature. As part of this review, I had intended to conduct a quick and dirty meta-analysis of the experimental literature to date (i.e., the current paper plus the five additional experiments listed below), but discovered that the authors of the current paper don't provide enough information to include their research in the meta-analysis. Page 17, where the authors present their well-being results, should include a table of means and standard deviations for the SWLS and SWEMWBS scores for Block 2 broken down by experimental condition. Table A-3, which only reports the regression coefficients and p-values does not provide sufficient information to reconstruct the needed information.

In summary, this is an interesting study with a laudable goal. Experimental evidence is hugely important, and the current study supplements other recent experiments restricting social media usage by throttling back usage over a 9-week period, compared to a maximum of four weeks in other work. However, the study has some important flaws that make me believe the paper shouldn't be published in its current form. The authors made arbitrary decisions about what online behavior to consider use of social media (i.e., only Facebook, Snapchat, and Instagram, but not WhatsApp), failed to differentiate types of use

(e.g., social interaction versus content consumption) even though the prior literature suggest that type of use is an important moderator, and was under powered. A more minor problem is that the authors don't provide enough information to allow their research to be used in a meta-analysis. I think the paper still has promise but needs to do more sophisticated data analysis (e.g., Instrumental variable approach mentioned previous or a mediation analysis) to link the changes induced by the experimental manipulation on well-being.

References

Allcott, H., Braghieri, L., Eichmeyer, S., & Gentzkow, M. (2020). The welfare effects of social media. *American Economic Review, 110*(3), 629-676.

Brailovskaia, J., Ströse, F., Schillack, H., & Margraf, J. (2020). Less Facebook use–More well-being and a healthier lifestyle? An experimental intervention study. *Computers in Human Behavior, 108*, 106332.

Hanley, S. M., Watt, S. E., & Coventry, W. (2019). Taking a break: The effect of taking a vacation from Facebook and Instagram on subjective well-being. *PloS one, 14*(6), e0217743.

Liu, D., Baumeister, R. F., Yang, C.-c., & Hu, B. (2019). Digital communication media use and psychological well-being: A meta-analysis. *Journal of Computer-Mediated Communication, 24*(5), 259-273.

Mosquera, R., Odunowo, M., McNamara, T., Guo, X., & Petrie, R. (2019). The economic effects of Facebook. *Experimental Economics*, 1-28.

Vanman, E. J., Baker, R., & Tobin, S. J. (2018). The burden of online friends: The effects of giving up Facebook on stress and well-being. *The Journal of social psychology, 158*(4), 496-508.
