# Supplementary material for: Effects of restricting social media usage on wellbeing and performance: A randomized control trial among students
Source: PLoS One. 2022 Aug 24;17(8):e0272416. doi: 10.1371/journal.pone.0272416 (PMC9401146; doi:10.1371/journal.pone.0272416)
Supplement: Attachment — Submitted filename: Response to Reviewers.pdf [file pone.0272416.s006.pdf]

Thank you for making the extensive changes to your paper in response to the reviewers' and my suggestions. The paper is much stronger but I have a few more suggestions before moving on to making a decision on publication.

Thank you for your helpful comments. We have incorporated a number of changes following your suggestions and believe we have addressed all your remaining concerns. Responses to your comments are listed below in blue.

First, a very real concern is the statistical power of your study, and I don't think your description of that problem on page 27 is very clear or helpful. First of all, we don't usually consider power from the point of view of the maximum difference we cannot detect. It would be easier to understand this if you talked about the minimum difference you can detect.

Thank you. We have reformulated this section and other sections of the paper to refer to the minimum detectable difference. In addition, we now clarify the power calculation for our study in the Sample section.

Second, I am puzzled as to why you are talking about increases in life satisfaction, when the concern is for decreases.

Thank you for pointing this out, we have reworded this section and we do not refer to increases in life satisfaction anymore.

Also, should you not be citing the differences you observed and discussing whether you could have found those to be significant with more power? That is what seems to be the issue.

Yes, we agree. We made the following edits based on your comment: First, we only mention how we calculate power for our study in the Sample section and drop our earlier discussion on maximum difference that we cannot detect (see also above response). Second, we now directly discuss what we observed and what we could have been significant with more power in the regressions subsection of the results section. We are open to making further edits if you think our discussion is not clear.

Given that you found a slight increase among the Android group, it might make sense to look at both groups separately in considering the power question.

Thank you for this comment. While we agree with it, we have not performed this analysis as an analysis for each of the groups would reduce the sample size and lower the power further. We added a footnote to propose this as a direction for future research.

I would also recommend discussing power in the method section with how large a difference you will be able to detect given the sample size you have. Then in the Results, you could comment on whether your differences are large enough to have rejected the null hypothesis with more power.

Thank you. As mentioned previously, we now directly address power in the Methodology (Sample) and Results sections in detail. We explain power calculation in the Sample section and comment on whether the differences we find are large enough to have rejected the null hypothesis with more power in the Results section.

I am also puzzled as to why you show the regressions for grades but not for wellbeing and life satisfaction in the Results. It seems to me that the mental health concerns are the ones that motivated this to begin with, so those findings should receive priority.

Thank you. We have now added regressions for well-being to the main text and now present them before the regressions of academic grades.

Finally, I think your findings could use more discussion than you devote to them. It seems that your intervention to reduce some forms of social media use served to increase other forms of interaction within one’s social network. So, what your findings seem to say is that social media may primarily serve as a way to connect with others and that arbitrarily defining those media as you have merely caused your students to migrate to other platforms that serve the same purpose. In other words, what your study shows is that with all of the ways that people can interact online, it is difficult to isolate some as more responsible for adverse effects. You would have to restrict the ability to use online platforms to communicate with others as a way to isolate that effect and I doubt that you could recruit a sample to do that for any length of time.

Thank you for nudging us to add more discussion. We now discuss how our definition of social media is just one way and future research can look at a broader set of apps

including instant messaging apps. We do not want to make the paper wordier, but we are happy to further extend the discussion if you think we are still missing something.

One other possible way that some social media may be harmful is by pushing advertising to users that they may not want exposure to and doing this to maintain continued engagement. This might be a mechanism for internet addiction that could be studied. In any case, I think a deeper discussion of these issues would be helpful for readers. Your point about the policy implications also follows from this, since many online media serve the same purpose and just differ in how they afford that opportunity.

We add this point to the discussion section.

I also think you have more findings to discuss, such as correlations between mental health and various uses of social and other media, such as in Table 4. Perhaps it is the video features of online media that are the source of adverse effects on mental health? And perhaps the sites push certain videos in order to maintain engagement?

Unfortunately due to the limitations of our data, we cannot observe what subjects do once they are on a social media app and what type of videos they watch on video streaming platforms. However, we add this as an avenue for future research in the discussion section.

In the abstract, I would describe the finding by saying that "we do not find any significant impact of social media usage as it was defined in this study on well-being…" As part of your limitations section, I would also take note of the fact that other forms of social media were not excluded in your study and this will be an obvious point of criticism.

Thank you for this comment. We have changed the abstract and added a part in the limitations section.

I also think it would be useful to consider a paper that just came in Clinical Psych Science by Sewall et al. They also find that various uses of digital tech are not very strongly related to mental health in young adults

Thank you for pointing out this new reference. We now added it to our literature review.
