# Supplementary material for: Effects of restricting social media usage on wellbeing and performance: A randomized control trial among students
Source: PLoS One. 2022 Aug 24;17(8):e0272416. doi: 10.1371/journal.pone.0272416 (PMC9401146; doi:10.1371/journal.pone.0272416)
Supplement: Attachment — Submitted filename: PONE-D-21-20386_R2[11].pdf [file pone.0272416.s007.pdf]

PLOS ONE

Effects of restricting social media usage on wellbeing and performance: A randomized control trial among students

--Manuscript Draft--

**Manuscript Number:**	PONE-D-21-20386R2
**Article Type:**	Research Article
**Full Title:**	Effects of restricting social media usage on wellbeing and performance: A randomized control trial among students
**Short Title:**	Effects of restricting social media usage
**Corresponding Author:**	Avinash Collis<br>The University of Texas at Austin<br>Austin, TX UNITED STATES
**Keywords:**	social media; Regulation; subjective well-being; performance; field experiment
**Abstract:**	Recent research has shown that social media services create large consumer surplus. Despite their positive impact on economic welfare, concerns are raised about the negative association between social media usage and well-being or performance. However, causal empirical evidence is still scarce. To address this research gap, we conduct a randomized controlled trial among students in which we track participants' daily digital activities over the course of three quarters of an academic year. In the experiment, we randomly allocate half of the sample to a treatment condition in which social media usage (Facebook, Instagram, and Snapchat) is restricted to a maximum of 10 minutes per day. We find that participants in the treatment group substitute social media for instant messaging and do not decrease their total time spent on digital devices. Contrary to findings from previous correlational studies, we do not find any significant impact of social media usage as it was defined in our study on well-being and academic success. Our results also suggest that antitrust authorities should consider instant messaging and social media services as direct competitors before approving acquisitions.
**Order of Authors:**	Avinash Collis
	Felix Eggers
**Opposed Reviewers:**	
**Response to Reviewers:**	Please see the attached document.
**Additional Information:**	
**Question**	**Response**
**Financial Disclosure**<br><br>Enter a financial disclosure statement that describes the sources of funding for the work included in this submission. Review the submission guidelines for detailed requirements. View published research articles from *PLOS ONE* for specific examples.<br><br>This statement is required for submission and **will appear in the published article** if the submission is accepted. Please make sure it is accurate.	A.C. received funding from the MIT Initiative on the Digital Economy for this research (https://ide.mit.edu/). There is no associated grant number. The funder had no role in study design, data collection and analysis, decision to publish, or preparation of the manuscript.<br><br>F.E. received funding from the Behavioral Research Lab at the Faculty of Economics and Business at the University of Groningen for this research (https://www.rug.nl/feb/?lang=en). There is no associated grant number. The funder had no role in study design, data collection and analysis, decision to publish, or preparation of the manuscript.

Powered by Editorial Manager® and ProduXion Manager® from Aries Systems Corporation

**Unfunded studies**<br>Enter: *The author(s) received no specific funding for this work.*<br><br>**Funded studies**<br>Enter a statement with the following details:<br>• Initials of the authors who received each award<br>• Grant numbers awarded to each author<br>• The full name of each funder<br>• URL of each funder website<br>• Did the sponsors or funders play any role in the study design, data collection and analysis, decision to publish, or preparation of the manuscript?<br>• **NO** - Include this sentence at the end of your statement: *The funders had no role in study design, data collection and analysis, decision to publish, or preparation of the manuscript.*<br>• **YES** - Specify the role(s) played.<br><br>* typeset	
**Competing Interests**<br><br>Use the instructions below to enter a competing interest statement for this submission. On behalf of all authors, disclose any competing interests that could be perceived to bias this work—acknowledging all financial support and any other relevant financial or non-financial competing interests.<br><br>This statement is required for submission and **will appear in the published article** if the submission is accepted. Please make sure it is accurate and that any funding sources listed in your Funding Information later in the submission form are also declared in your Financial Disclosure statement.<br><br>View published research articles from *PLOS ONE* for specific examples.	The authors have declared that no competing interests exist.

**NO authors have competing interests**<br><br>Enter: *The authors have declared that no competing interests exist.*<br><br>**Authors with competing interests**<br><br>Enter competing interest details beginning with this statement:<br><br>*I have read the journal's policy and the authors of this manuscript have the following competing interests: [insert competing interests here]*<br><br>* typeset	
**Ethics Statement**<br><br>Enter an ethics statement for this submission. This statement is required if the study involved:<br><br>• Human participants<br>• Human specimens or tissue<br>• Vertebrate animals or cephalopods<br>• Vertebrate embryos or tissues<br>• Field research<br><br>Write "N/A" if the submission does not require an ethics statement.<br><br>General guidance is provided below. Consult the submission guidelines for detailed instructions. **Make sure that all information entered here is included in the Methods section of the manuscript.**	The study received approval from the Institutional Review Board of the University of Groningen. The approval number is FEB-20180903-7314. Written informed consent was obtained from the subjects.

Powered by Editorial Manager® and ProduXion Manager® from Aries Systems Corporation

**Format for specific study types**<br><br>**Human Subject Research (involving human participants and/or tissue)**<br>• Give the name of the institutional review board or ethics committee that approved the study<br>• Include the approval number and/or a statement indicating approval of this research<br>• Indicate the form of consent obtained (written/oral) or the reason that consent was not obtained (e.g. the data were analyzed anonymously)<br><br>**Animal Research (involving vertebrate animals, embryos or tissues)**<br>• Provide the name of the Institutional Animal Care and Use Committee (IACUC) or other relevant ethics board that reviewed the study protocol, and indicate whether they approved this research or granted a formal waiver of ethical approval<br>• Include an approval number if one was obtained<br>• If the study involved *non-human primates*, add *additional details* about animal welfare and steps taken to ameliorate suffering<br>• If anesthesia, euthanasia, or any kind of animal sacrifice is part of the study, include briefly which substances and/or methods were applied<br><br>**Field Research**<br><br>Include the following details if this study involves the collection of plant, animal, or other materials from a natural setting:<br>• Field permit number<br>• Name of the institution or relevant body that granted permission	
**Data Availability**<br><br>Authors are required to make all data underlying the findings described fully available, without restriction, and from the time of publication. PLOS allows rare exceptions to address legal and ethical concerns. See the PLOS Data Policy and FAQ for detailed information.	No - some restrictions will apply

A Data Availability Statement describing where the data can be found is required at submission. Your answers to this question constitute the Data Availability Statement and **will be published in the article**, if accepted.<br><br>**Important:** Stating 'data available on request from the author' is not sufficient. If your data are only available upon request, select 'No' for the first question and explain your exceptional situation in the text box.<br><br>Do the authors confirm that all data underlying the findings described in their manuscript are fully available without restriction?	
**Describe where the data may be found in full sentences. If you are copying our sample text, replace any instances of XXX with the appropriate details.**<br><br>• If the data are **held or will be held in a public repository**, include URLs, accession numbers or DOIs. If this information will only be available after acceptance, indicate this by ticking the box below. For example: *All XXX files are available from the XXX database (accession number(s) XXX, XXX.).*<br>• If the data are all contained **within the manuscript and/or Supporting Information files**, enter the following: *All relevant data are within the manuscript and its Supporting Information files.*<br>• If neither of these applies but you are able to provide **details of access elsewhere**, with or without limitations, please do so. For example:<br><br>*Data cannot be shared publicly because of [XXX]. Data are available from the XXX Institutional Data Access / Ethics Committee (contact via XXX) for researchers who meet the criteria for access to confidential data.*<br><br>*The data underlying the results presented in the study are available from (include the name of the third party*	Raw data cannot be shared publicly since it is protected under GDPR. The data was collected at the University of Groningen and contains sensitive data including all online activities of students and their grades. The IRB of the University of Groningen did not give the authors permission to share raw data. Please contact privacy@rug.nl if you have any questions regarding this. If you receive approval from the privacy office at the University of Groningen (privacy@rug.nl), we will share the raw data with you.<br><br>Minimal data used to replicate the study's main results are provided at: https://osf.io/jfqp3/ (DOI 10.17605/OSF.IO/JFQP3)

and contact information or URL).<br>• This text is appropriate if the data are owned by a third party and authors do not have permission to share the data.<br><br>* typeset	
Additional data availability information:	Tick here if the URLs/accession numbers/DOIs will be available only after acceptance of the manuscript for publication so that we can ensure their inclusion before publication.

Dear Dr. Romer

Thank you for providing us with the opportunity to again revise our submission and for your helpful comments. We have incorporated a number of changes following your suggestions and believe we have addressed all your remaining concerns. We are happy to make further edits if you have any additional comments.

Please let us know if you have any questions. We look forward to hearing from you soon.

Avinash Collis & Felix Eggers

Manuscript

Click here to access/download;Manuscript;Manuscript with formatted citations.docx

Effects of restricting social media usage on wellbeing and performance: A randomized control trial among students

Avinash Collis[1*¶], Felix Eggers[2*¶]

[1] McCombs School of Business, The University of Texas at Austin, Austin, Texas, USA

[2] Copenhagen Business School, Frederiksberg, Copenhagen, Denmark

*Corresponding authors

Email: avinash.collis@mccombs.utexas.edu (AC), fe.marktg@cbs.dk (FE)

[¶]These authors contributed equally to this work.

Abstract

Recent research has shown that social media services create large consumer surplus. Despite their positive impact on economic welfare, concerns are raised about the negative association between social media usage and well-being or performance. However, causal empirical evidence is still scarce. To address this research gap, we conduct a randomized controlled trial among students in which we track participants' daily digital activities over the course of three quarters of an academic year. In the experiment, we randomly allocate half of the sample to a treatment condition in which social media usage (Facebook, Instagram, and Snapchat) is restricted to a maximum of 10 minutes per day. We find that participants in the treatment group substitute social media for instant messaging and do not decrease their total time spent on digital devices. Contrary to findings from previous correlational studies, we do not find any significant impact of social media usage as it was defined in our study on well-being and academic success. Our results also suggest that antitrust authorities should consider instant messaging and social media services as direct competitors before approving acquisitions.

Introduction

Social media increasingly plays an important role in our daily lives. Ever since the launch of major modern social media platforms such as Facebook, users have adopted them at an explosive pace and adoption continues to increase to this day. Almost 3 billion users are active monthly on Facebook in 2021 [1]. This corresponds to over a third of the global population. The average adult spends over 45 minutes every day on social media platforms [2].

Given this rapid adoption and usage of social media platforms, it is essential to study the impact of social media on the well-being of users. Brynjolfsson et al. [3] find that digital technologies, including social media, generate a large amount of consumer surplus. More specifically, they conduct incentive compatible choice experiments to measure the consumer surplus generated by Facebook and find that the median US Facebook user obtains around $48/month of value from using Facebook in 2017 as measured from their willingness to accept to give up access to Facebook for a month. They also conduct a similar experiment with students at a large European university and find that the median student in their sample obtains €97/month of value from using Facebook.

While Facebook and other social media services seem to generate a large amount of consumer surplus and contribute towards the economic well-being of their users, questions are raised about the negative externalities generated by social media. There is an active debate in media and academic research about the impact of social media on subjective well-being (including happiness and life satisfaction) and productivity. Current empirical results are ambiguous. Across different studies, correlational evidence points towards a positive, neutral (null results) and negative relationship between social media use and well-being (see Haidt [4] for a comprehensive literature review of social media use and mental health). However, most of this evidence suffers from issues related to reverse causality [5] and inaccurate measures of self-reported social media use [6]. Rigorous causal evidence on long term impacts of social media use on well-being is lacking.

Concerns are also raised in the field of Education policy on the impact of screen time (including social media use) on academic performance of students. Critics contend that social media use on smartphones distracts students from focusing in classes and affects their grades. Motivated by these concerns, the French education ministry banned smartphones in schools from first through ninth grades [7]. The American Academy of Pediatrics also recommends

parents to limit the time spent by children and adolescents on social media so that they have enough time left to study [8]. However, a rigorous analysis of the data used in previous correlational studies that were used as evidence to support these policies suggests that the effects of social media use and screen time on adolescent well-being are too small to warrant policy changes [9].

Given these widespread concerns and conflicting correlational evidence on the impact of social media on well-being, it is necessary to obtain causal evidence in a timely manner before policies are implemented hastily. We seek to fill this research gap by conducting a randomized controlled trial to measure the causal long-term impact of social media use on academic performance and well-being. Specifically, the research question we aim to answer is: How does restricting social media use affect a) well-being, b) academic performance, and c) other digital activities?

We recruit students at a large European university to be part of our study over the course of three academic terms (quarters). The subjects install a software (RescueTime) on their personal computers and mobile devices. This software tracks all of the digital activities of the subjects during the entire duration of the study period. The first term serves as the baseline period. In the second term, subjects are randomized into treatment and control groups and the treatment group has social media use (Facebook, Instagram and Snapchat) restricted to a maximum of 10 minutes per day across all devices. We then measure the post-treatment effects in the third term.

We observe the entire space of digital activities performed by our subjects that covers online and also offline activities on their devices, including activities related to learning (such as writing in Microsoft Word or reading a PDF). Our social media use metrics are computed based on the actual time spent on social media and are not based on self-reported metrics of time spent, which is predominantly used in the existing literature. In addition to the digital

activities, we obtain objective metrics of performance (grades) and subjective well-being scores solicited through surveys.

Contrary to results from previous studies using observational data, we do not find evidence that social media causes a positive or negative impact on well-being (including life satisfaction and mental health). Moreover, we also do not find any evidence that social media usage impacts academic success. However, we find significant substitution effects. Specifically, we see that participants in the treatment group substituted their use of social media services for instant messaging apps (e.g. WhatsApp). In total, these participants do not spend less time on their digital devices (computers and mobile phones) as those in the control group.

Our paper makes three main contributions. First, we test the popular media narrative portraying social media as the villain responsible for negatively affecting well-being of society. We do not find any evidence supporting this hypothesis. Second, educators and parents are increasingly concerned about the impact of digital distractions on academic performance and are restricting the online activities of students (for example through parental control software or by taking away their devices). While previous evidence seems to suggest that device usage in class might negatively affect academic performance, our results show that restricting social media usage from the lives of students (inside and outside class) might not have the intended effect. Finally, our paper is one of the first to provide evidence of substitutability between social media and instant messaging apps. This has major implications for antitrust authorities analyzing the market power of major social media platforms such as Facebook which owns Instagram (another social media service) and WhatsApp (instant messaging service).

The paper proceeds as follows. In the next section, we provide a brief review of existing literature on the impact of social media use on well-being and academic performance. In the

following section, we describe the design of our experiment and data collected over the course of the study. We then show the main results and conclude with a discussion of the limitations of this study and directions for future research.

Related Literature

The impact of the internet in general, and social media in particular, on well-being has attracted the attention of a number of researchers in the fields of psychology, epidemiology and human-computer interaction (HCI) over the past two decades. Most of this literature uses self-reported metrics of technology use and provides cross-sectional correlational evidence. Kraut and Burke [10] provide a review of this literature and express skepticism regarding cross-sectional and survey-based studies due to the presence of several confounding factors. Moreover, correlational studies might suffer from an abundance of researcher degrees of freedom and the file drawer problem such that only significant results are published, inevitably leading to the implication that social media usage either has a positive or negative effect. However, a null result or insignificant findings regarding social media usage might be a plausible outcome.

Orben and Przybylski [9] rigorously analyze popular large scale social datasets (n=350k) used in previous correlational studies studying the impact of technology use on well-being by conducting a specification curve analysis of the data. This analysis involves running all possible analytical models using various combinations of the covariates. Instead of selective reporting, results from all of these analyses are reported. They find a small negative association between digital technology use and adolescent well-being. However this effect is economically insignificant explaining at most 0.4% of the variation in well-being. For comparison, the authors show that seemingly neutral activities such as eating potatoes have the same negative association with well-being as technology use. Given these concerns with correlational analyses involving cross sectional data, Kraut and Burke [10] call for

experimental evidence paired with tracking data to provide reliable evidence on the relationship between internet use and well-being.

The subset of literature focusing on the association between social media use and well-being has found a wide range of effects (negative, mixed, positive and null). Using a longitudinal survey, Shakya and Christakis [11] found a negative association between Facebook use and well-being. In contrast, Burke et al. [12] find a positive association between directed communication on Facebook and social well-being due to subjects reporting improved feelings of social bonding and reduced loneliness. Similarly, Hobbs et al. [13] match Facebook profiles with public health records and find that being more socially integrated online (by accepting more Facebook friends) is associated with reduced risk of mortality. Burke and Kraut [14] find that targeted messages from strong ties is associated with positive improvements in well-being while viewing messages from friends broadcasted to all of their friends and receiving one-click feedbacks were not associated with any improvement in well-being. Kim and Shen [15] find that directed communication activities on social media platforms are positively associated with life satisfaction for older users.

Schemer et al. [16] and Johannes et al. [17] do not find any substantial relationship between social media use and subjective well-being. Liu et al. [18] conduct a meta-analysis of 124 studies and find that the association between social media use and well-being depends on the type of social media use. This association is positive for social interactions and negative for content consumption. Huang [19] also conducted a meta-analysis of 61 studies and documented a weak correlation between time spent on social media and negative indicators including depression and loneliness. For a comprehensive list of all studies studying social media use and well-being, see literature reviews by Appel et al. [20], Meier and Reinecke [21], Dienlin and Johannes [22], Masur [23] and Orben [24]. Cheng et al. [5] combine a survey of Facebook users with their Facebook activities and find that subjects reporting

problematic use of Facebook were also going through a major life event such as a breakup. This shows that confounding variables could be a major concern in previous studies associating social media use and well-being.

Orben et al. [6] use a large-scale longitudinal dataset and conduct a specification curve analysis to rigorously analyze the relationship between adolescent social media use and well-being. Most of the analyses report tiny, trivial and insignificant results. Moreover, they provide evidence for reverse causality showing that social media use predicts well-being in the future and vice versa. Similarly, Sewall et al. [25] also find little to no evidence that changes in social media or smart phone use predict psychological distress.

Another major concern related to existing studies is the use of self-reported usage data. Survey respondents are typically asked to report the average time they spend on the internet, social media and digital devices. Several papers show that self-reported measures of technology use (including social media usage) are poorly correlated with actual usage and contain systematic patterns of misreporting [26-29].

Given this inconclusive evidence and lack of objective technology use data in existing literature, it is essential to obtain reliable causal evidence in a timely manner to inform policy makers. We aim to resolve this gap by obtaining evidence through a randomized controlled trial and using objective technology use metrics tracked by a software installed on the digital devices of our experimental subjects. In terms of outcome variables, we track measures of subjective well-being (life satisfaction and mental health) and performance (grades and number of credit points) over the duration of three quarters of an academic year (8 months) with the actual treatment lasting 2.5 months.

There is a related stream of literature using experiments to study the relationship between social media or computer usage and well-being or performance. Verduyn et al. [30] conduct a lab experiment where subjects are primed to passively use Facebook for 10 minutes and find

that passive use is associated with a decline in subjective well-being. However, it is not straightforward if results from a 10-minute treatment can be generalized to long term effects. Marotta and Acquisti [31] conduct an experiment with workers recruited from Amazon mechanical turk and offer productivity enhancing tools to subjects. One of the treatment groups has popular social media sites blocked during work hours. They find that workers in this group completed more tasks and increased their earnings. Carter et al. [32] conduct a randomized controlled trial in a US university where classes in the treatment group prohibited the use of computers in the class. They find that average exam scores were higher in the treatment group compared to the control group classes where students were allowed to use their computers. Using causal inference methods on observational data, Belo et al. [33] and Beland and Murphy [34] study the impact of broadband access and banning mobile phones in schools respectively on academic performance and also find evidence suggesting that digital distractions during class reduce academic performance. Taken together, evidence seems to suggest that digital device use in class or at work is harmful for student or worker performance. However, the overall causal impact of social media usage in life (inside and outside class or at work) on performance and well-being still remains an open question. Our study complements this research by analyzing the overall long-term impact of social media on well-being and performance as the subjects in our treatment group has restricted use of social media throughout their day for a long period of time.

A study closely related to our research is the experiment conducted by Allcott et al. [35]. They conducted a randomized controlled trial of Facebook users where subjects in the treatment group had to deactivate their Facebook account for 1 month. They find that this treatment reduced total online activity including other social media and this reduction persists after the end of the experiment. However, they use self-reported metrics of usage of online activities which are weakly correlated to objective usage metrics according to previous

research. They measure 11 different metrics of subjective well-being and find that deactivating Facebook led to increase in subjective well-being for 4 out of the 11 metrics. Other related experiments include Brailovskaia et al. [36] (two weeks intervention, self-reported Facebook usage measure), Hall et al. [37] (one to four weeks intervention, self-reported Facebook usage measure), Hinsch and Sheldon [38] (one week intervention, self-reported Facebook usage measure), Tromholt [39] (one week intervention, self-reported Facebook usage measure), Mosquera et al. [40] (one week intervention), Hanley et al. [41] (one week intervention) and Vanman et al. [42] (five days intervention). Overall, the magnitude of the effects is small and it is not clear if these effects would have persisted for a treatment of longer duration. For a longer treatment duration, subjects could learn to live in a world without Facebook by discovering alternative substitutes providing similar use cases and their subjective well-being scores could go back to pre-experiment levels.

Methods

Experimental Procedure

We recruited students in the faculty of economics and business of a large European university to take part in an academic study. The study received approval from the Institutional Review Board of the university. We used a flyer to invite students in lectures and from the pool of participants of the behavioral research lab of the university. The flyer informed students about the subject of the study, the required activities, the reward, and about measures to protect the participants' privacy. Specifically, we let the students know that the study required to install a software (RescueTime) on their computers and mobile devices that keeps track of their digital activities and that allows them to analyze how much time they are spending on various categories of activities. We also stated that the study tracks their academic performance and well-being. Moreover, we informed the students that, in order to

qualify for the reward, they need to keep the software running during the time of the study and to take part in four online surveys; one at the beginning of the study and one after each quarter. In addition to getting the software for free, we offered students €20 and a one out of 100 chance to win €1,000 if they take part until the end of the study.

The sign-up link forwarded interested students to a registration form that provided a more detailed privacy statement (about aim and principles of processing personal data, the types of data used, the limited recipients of the data, the storage period, and the students' rights), informed consent, and asked students for their student email address, basic information about their studies (program, year), and the number and type of computers and mobile devices. The registered students were then invited to the study according to the experimental design detailed below.

Experimental Design

The recruitment of students took place in the first quarter of the academic year 2018/19. We scheduled the experiment to run for the remaining three academic quarters. We will refer to these three terms as block 1, 2, and 3 of the study (which are quarter 2, 3, and 4 of the academic year). Each block consists of seven weeks of teaching and two examination weeks. The specific timeline was:

- Block 1: from mid-November to end of January, with holidays from December 24 to January 3.
- Block 2: from February to mid-April.
- Block 3: from mid-April to end of June, with holidays from April 19 to 24.

We used the first block to establish a baseline of the students' digital activities. In block 2, we randomly assigned participants to one of two conditions: a control group without specific instructions and a treatment group that received an incentive to use social media as little as possible. Specifically, we instructed them to use Facebook, Instagram and Snapchat for a

maximum of 10 minutes per day. We did not block these services completely because not having access to social media at all might have a negative effect on students, e.g., if they use it to exchange important information about their studies. The 10-minute limit enables students to still access relevant information while not allowing them to waste a longer period of time. This is consistent with the Goldilocks hypothesis according to which moderate digital use may be advantageous compared to no use or overuse [43]. The software would inform students in the treatment group when they reached the limit and automatically block Facebook, Instagram and Snapchat afterwards. Students could disable this feature if they needed to use these services for longer. We informed students that we gave away another €1,000 among all students who achieved to stay under the 10-minute limit throughout block 2. Block 3 served to assess post-treatment effects.

We focus on Facebook, Instagram, and Snapchat in our experiment because they were the most popular social media services according to our measurement in block 1. As a caveat, the distinction of these services from others such as instant messaging can be debated. We return to this issue in the discussion.

We invited students to four surveys in total. We have sent the first survey in the first week of block 1. This survey asked students to give informed consent and, after referring them to the privacy statement, their agreement to use their academic grades for the purpose of the study. Moreover, we asked them about basic demographic information, their study program, and additional work activities next to their studies. Moreover, we provided measures of subjective well-being (see specific measures below). Upon completion of this first survey, we gave students the installation and registration instructions for the tracking software and asked them to keep this software running henceforth on all their computers and mobile devices. While the software was supported by Windows, OS X, and Android devices, it was not compatible with iOS devices (iPhone or iPad). In order to make sure that students with iOS devices complied

to the 10 minute limit in the treatment condition, we informed them that we will ask them at a random time to hand in a screenshot of the Screen Time feature of iOS that reports similar information.

Surveys 2, 3, and 4 followed after each block and repeated the subjective well-being measures in order to track students' well-being over time. We gave students a one-week deadline to fill out each survey.

Survey Measures

As measures of subjective well-being we use the satisfaction with life scale (SWLS) [44] that consists of five items (In most ways my life is close to my ideal; The conditions of my life are excellent; I am satisfied with my life; So far I have gotten the important things I want in life; If I could live my life over, I would change almost nothing). These items are measured on a 7-point scale (1 "strongly disagree" to 7 "strongly agree"). SWLS is the most widely used scale to measure subjective well-being and is also used in previous studies studying social media use and well-being (e.g., [30, 45]). In addition to SWLS, we also collected direct measures of happiness and life satisfaction through standard questions widely used in previous literature. Besides numerous other studies, the happiness question is used in the World values survey [46] and the life satisfaction question is used by Gallup [47] to calculate its well-being index. These questions are highly correlated with objective measures of well-being such as brain activity, emotional expressions and suicide rates as well as decision utility [48]. We obtain qualitatively similar results using these happiness and life satisfaction scores as we found using SWLS.

For measuring mental well-being, we adopted the shortened Warwick-Edinburgh Mental Well-being Scale (SWEMWBS) [49, 50] with seven items (I've been feeling optimistic about the future; I've been feeling useful; I've been feeling relaxed; I've been dealing with problems well; I've been thinking clearly; I've been feeling close to other people; I've been

able to make up my own mind about things). These items are assessed on a 5-point scale ranging from “None of the time” to “All of the time”. The SWEMWBS is a popular scale to measure mental well-being and is used in previous studies studying technology use and mental well-being [43].

Overview of Data Sources

Overall, our study makes use of three data sources: digital activities tracked by the software, self-reported measures via surveys, and academic grades from the educational administration. Table 1 shows an overview of these data types.

The software tracks users’ activities on each device in 5-minute intervals and records how many seconds a user has actively used a specific program, app, or website in this interval, ranging from 1 to 300 seconds. Specifically, it records the user id, the name of the activity, the system name (Windows, Mac OS, or Android), and a timestamp. Since we are specifically interested in social media activities of the three most used social network services Facebook, Instagram and Snapchat, we used lookup tables to classify activities accordingly. For example, Facebook usage could appear in the activities as “facebook.com”, “fb.com”, “messenger.com”, “Facebook for Android”, “Facebook for Windows”, etc. We gathered this list of activities in block 1 and used each of these activities to count toward the 10-minute limit for the treatment group in block 2.

The European university at which this study took place uses a grading system that ranges from 0 to 10. Any grade below 6 represents a fail. A grade of 7 is most common and often referred to as “standard”, a 6 as “below standard“, and an 8 or higher as “above standard”. The grade for a lecture typically consists of a combined grade of the final exam and assignments that have to be completed during the course.

Table 1: Overview of data sources

Type	Measure	Source	Data collection
Digital activities	Usage in number of seconds	Tracked by software	On each participant's device throughout the entire study
Subjective well-being	Rating scales	Self-reported in surveys	At the beginning of the study and after each teaching block
Academic grades	From 0 to 10, with <6 = failed, 6 = below standard, 7 = standard, >8 above standard;	Educational administration	Once at the end of the academic year

Sample

A total of 191 respondents completed the first survey. As is typical for longitudinal studies, some students dropped out over time such that 157 students completed survey 2, 144 survey 3, and 121 the final survey. The survey participation corresponds to the number of participants who reported digital activities using the software (see Table 2).

The following results will be based on the sample that recorded activities for at least 30 days in block 1 and 2 and completed surveys 1, 2, and 3. We will analyze the post-treatment data from block 3 and survey 4 separately. From the 134 students who recorded activities in block 1 and 2, we were able to match 122 from all data sources, i.e., twelve students did not answer (one of) the surveys or did not follow courses in at least one of the blocks.

Despite the dropouts, most importantly, there are no significant differences between the treatment and the control group, in terms of gender (Chi-squared test, $p = 0.471$), age (t-test, $p = 0.961$), mobile device operating system (Chi-squared test, $p = 1.000$), number of years studying at the university (t-test, $p = 0.334$) or whether students are working next to their studies in block 1 (Chi-squared test, $p = 0.974$) or block 2 ($p = 0.594$) (see Table 3 for details). There are also no significant differences between those who started the study and those who dropped out in terms of gender (Chi-squared test, $p = 0.701$), age (t-test, $p = 0.113$), mobile operation system (Chi-squared test, $p = 0.975$), of work status in block 1 (Chi-squared test, $p = 0.109$) or block 2 ($p = 0.169$). However, there is a significant difference between these samples regarding the study year (t-test, $p = 0.027$) such that those who

dropped out are more likely to be Bachelor degree students than Master's students. One potential explanation is that Bachelor degree students are more likely to quit their studies and not have any courses or grades registered subsequently.

For the subsequent analysis, we note that at 80% power and alpha = 5%, the minimum detectable difference with our sample size is 2.5 for SWLS (assuming a standard deviation of 5.0), 1.5 for SWEMWBS (standard deviation = 3.0), and 0.5 for academic grades (standard deviation of 1.0).

Table 2: Number of participants

Part	Number
Completed survey 1	191
Used software in block 1 (calibration)	149
Completed survey 2	157
Used software in block 2 (treatment)	134
Completed survey 3	144
Used software in block 3 (post-treatment)	125
Completed survey 4	121
Took courses in block 1, 2, and 3	158

Table 3: Descriptive statistics of the sample

	Treatment	Control
Number of students	60	62
Gender: Female (vs. male)	0.467	0.548
Age (SD)	22.1 (3.3)	22.1 (3.1)
Mobile device operating system: Android (vs. iOS)	0.500	0.500
Studying in first to third year (vs. more than three years)	0.667	0.629
Working next to studying (block 1)	0.400	0.419
Working next to studying (block 2)	0.500	0.435

Results

Digital Activities

Social media usage

On average, students tracked 223.7 minutes of digital activities per day across the entire study (SD = 115.1 minutes). Students who use an Android mobile device recorded significantly more activities (265.6 minutes; t-test, $p < 0.001$), compared to students with an iOS device (182.5 minutes) as iOS was not supported by the software. While our activity estimates are more accurate for Android users we expect the treatment condition to be equally effective for both of these segments because we informed participants to also inspect their iOS tracked activities (see above).

Figure 1 shows the total number of minutes tracked by day, averaged for students with Android mobile devices in the treatment (black dots) and control groups (white dots). We report the activities for users with Android devices because the tracking is more accurate as it captures activities on desktop/laptop computers and their mobile devices (figures corresponding to the overall sample are in the Supporting Information, Figure S1). The solid vertical lines separate blocks 1, 2, and 3 and the dashed vertical lines indicate the start of the examination period. Overall, digital activities remain on a high level each day but are reduced during the winter holiday season and during the examination periods.

As a manipulation check, the bottom part of Figure 1 shows activities for social networking (Facebook, Instagram, and Snapchat combined) for the Android sample. The mean daily usage in minutes is 21.1 minutes (27.9 minutes) for users in the treatment (control) group in block 1, which is not significantly different (t-test, $p = 0.310$). The incentive to reduce social media activities was effective as students in the treatment condition significantly reduced their social media usage in block 2 compared to the control group (t-test, $p = 0.009$). The

horizontal line represents the 10-minute limit imposed on the treatment group. The average usage per day is close to the limit in the treatment group with 8.1 minutes. Within the control group, the average daily usage of 24.2 minutes in block 2 is on the same level as in the first block (paired-sample t-test, p = 0.245).

Figure 1: All digital activities and social media usage over time (users with Android devices)

Remarkably, although students in the treatment group significantly reduced their social media activities, their overall digital activities overall are not affected but, in fact, exceed those of the control group in block 2 (t-test, p = 0.026). This result indicates that students substituted or even overcompensated their social media usage with other activities.

Substitution. Figures 2.1 and 2.2 show the time series of activities of users with an Android device for the most used categories of services (we exclude the categories of general utilities, which holds mostly operating system activities, and uncategorized services for which there are no significant differences between the groups; activities for all users are in the Supporting Information, Figures S2.1 and S2.2). We find significant substitution for social networking with instant messaging (p = 0.008). Accordingly, more students in the treatment condition used instant messaging in block 2 when social media was restricted compared to block 1 and to the control group (difference-in-differences). The activities increased from an average daily use of 25.1 minutes in block 1 to 28.8 minutes in block 2 in the treatment group, while the usage decreased from 21.8 minutes to 15.2 minutes in the control group. Most activities (92.9%) in this category are related to WhatsApp.

We also find a significant increase in usage of music for Android users (t-test, p = 0.027) in the treatment group in block 2. However, average daily activities in this category are rather

low (below five minutes) and the difference is mostly driven by two outliers who listen to music for more than 30 minutes each day on average. Other activities show plausible patterns, e.g., the reference and learning category (activities include the university intranet, PDF reader, Wikipedia, Mendeley, Google scholar, EBSCO, etc.) shows peaks before the exam period. However, these and other activities are not affected by reduced social media usage (an overview of significance tests comparing treatment and control groups in block 2 vs. block 1 is given in the Supporting Information, Table S1).

Figure 2.1: Tracked digital activities over time (users with Android devices)

Figure 2.2: Tracked digital activities over time (users with Android devices)

Subjective Well-being

For the subjective well-being measures, the SWLS and SWEMWBS items show high reliability (Cronbach's α being 0.84 and 0.76 respectively). For the analysis we calculate scores as the sum of their items (with SWEMWBS being transformed according to a defined conversion table). The students score averages (SD) in SWLS of 25.0 (5.5), 25.0, (5.4), 25.1 (5.3) in the three surveys at the beginning of the study and after block 1 and 2. That means they are between an "average" and "high" score of satisfaction. Treatment and control group are not significantly different at the beginning of block 1 (t-test, p = 0.182; survey 1), at the end of block 1 (t-test, p = 0.212; survey 2), or, most importantly, at the end of block 2 after the exposure to the treatment (t-test, p = 0.167; survey 3). The same implications hold for the SWEMWBS scores that shows average scores (SD) of 22.8 (3.0), 22.5 (2.7), and 22.4 (3.3) in the three surveys (see Table S2 in the Supporting Information for details).

Figure 3 plots the differences between survey 3 and survey 2 (before and after the social media restriction) in terms of SWLS and SWEMBS. The distributions are centered on zero, illustrating the non-significant difference between the treatment and control group.

Figure 3: Differences in subjective well-being measures

Table 4 shows correlations with the subjective well-being measures and the digital activities in block 1 (i.e., activities that are not affected by the treatment condition) for users with an Android device. We detail the correlations of the subjective well-being measures at the beginning of the first block (in survey 1) and at the end of the block (in survey 2) to study potential reverse causality, e.g., increased well-being leads so more/less social media activities or vice versa.

Satisfaction with life and mental well-being are positively correlated and are also significant predictors over time, i.e., subjective well-being in survey 1 is positively correlated with the same measure in survey 2. Regarding digital activities, we see, on average, negative correlations between subjective well-being and all digital activities, albeit not being significant. Similarly, we do not find significant correlations with social media use. (We will address causality in the section below.) To address potential non-linear effects we also report correlations with categories of social media usage. Specifically, we used dummy variables relating to low usage with an average of less than 2 minutes per day (36.1% of users), medium usage of 2 to 20 minutes (39.5%), and high usage of 20 minutes or more (24.4%). A non-linear relationship is likely as low usage generally shows the most negative subjective well-being scores, while medium usage and not high usage scores the highest well-being. However, we cannot rule out reverse causality regarding these findings as the only significant

relationships are between satisfaction with life measured in survey 1 and the social media activities measured after the survey has taken place.

Activities related to communication, i.e., instant messaging and email, show significant positive correlations in the second surveys (for instant messaging regarding mental well-being and for email in terms of satisfaction with life). A consistent significant negative correlation can be observed for activities in the video category and satisfaction with life (both surveys) and mental well-being (survey 1). This suggests that less satisfied students and those with lower mental well-being at the beginning of the block increasingly watch videos in the subsequent block.

Table 4: Correlations of subjective well-being measures and digital activities in block 1 (Android users)

	SWLS Survey 1 (start of block 1)	SWLS Survey 2 (end of block 1)	SWEMWBS Survey 1 (start of block 1)	SWEMWBS Survey 2 (end of block 1)
SWLS Survey 2 (end of block 1)	**0.80**			
SWEMWBS Survey 1 (beginning of block 1)	**0.76**	**0.63**		
SWEMWBS Survey 2 (end of block 1)	**0.63**	**0.72**	**0.77**	
All digital activities	-0.06	-0.11	-0.15	-0.05
Social media usage	-0.03	-0.02	-0.13	-0.02
Social media usage low	**-0.31**	-0.23	-0.08	-0.13
Social media usage medium	**0.26**	0.19	0.14	0.15
Social media usage high	-0.02	-0.01	-0.08	-0.05
General Reference & Learning	-0.13	-0.14	-0.20	-0.06
Instant Message	0.18	0.24	0.14	**0.27**
Browsers	-0.09	-0.13	-0.01	-0.11
Video	**-0.28**	**-0.27**	**-0.28**	-0.21
Writing	0.24	**0.27**	0.10	0.09
Search	-0.19	-0.19	-0.22	-0.10
Email	0.21	**0.28**	0.11	0.10
General News & Opinion	0.19	0.04	0.14	0.13

Games	0.06	-0.17	0.04	-0.04
Presentation	0.14	0.10	-0.01	0.03
General Shopping	-0.05	0.02	-0.12	0.02
Music	0.12	0.10	0.12	0.24

(Correlations in bold font are significant on a 5% level)

Academic Performance

The students participating in our study scored an average (SD) of 7.105 (1.078) in block 1 and 7.122 (0.954) in block 2. Most grades can be classified as “standard”. The average (SD) sum of credit points per block is 13.571 (5.754) in block 1 and 12.353 (5.118) in block 2. Differences between the treatment and control group are not significant for grades (t-test, $p = 0.113$) but for the number of credit points such that the treatment group attempted to score significantly more credits (t-test, $p = 0.035$). This is visualized in Figure 4 as the difference in grades and credits in block 1 and 2. Note that the number of ECTS represents the courses that the student *attempted* to pass but they are also stored if the student failed the exam. A comparison of the number of successfully passed courses shows no significant differences between the groups (t-test, $p = 0.383$).

Figure 4: Differences in academic performance

Table 5 shows the correlations of academic performance in block 1 with the subjective well-being measures and digital activities. Accordingly, grades in block 1 are positively related to grades in block 2. The grades are not significantly correlated with the number of credit points, possibly due to a trade-off regarding a good grade and completing more courses. Credits are also not positively related over time, which is reasonable as more credits in one term means that the students have to obtain fewer credits in subsequent terms. The average

grade is positively and significantly correlated with satisfaction with life measures. This holds for SWLS measures at the beginning and end of the block.

Regarding correlations with digital activities we can observe significant positive effects on the average grade for writing and presentation activities, which are required to complete assignments (that are part of the grade for the majority of courses). We also see a positive effect of instant messaging on the grade, however, a negative effect on the number of credit points. Social media usage is not significantly correlated with the academic performance in block 1. Only when categorizing students based on their social media usage we see significant effects such that medium usage is negatively correlated with the number of credits.

To what extent these findings can be interpreted as causal evidence will be addressed in the following section by analyzing the complete randomized control trial across the two blocks using difference-in-differences analyses with regression models.

Table 5: Correlations of academic performance with measures of subjective well-being and digital activities in block 1 (Android users)

	Average grade in block 1	Sum of credit points in block 1
Sum of credit points in block 1	-0.16	
Average grade in block 2	**0.46**	-0.19
Sum of credit points in block 2	0.06	0.01
SWLS Survey 1 (beginning of block 1)	**0.27**	-0.10
SWLS Survey 2 (end of block 1)	**0.31**	0.02
SWEMWBS Survey 1 (beginning of block 1)	0.16	-0.07
SWEMWBS Survey 2 (end of block 1)	0.22	0.01
All digital activities	0.16	-0.16
Social media usage	0.02	-0.03
Social media usage low	-0.08	0.21
Social media usage medium	0.06	**-0.26**
Social media usage high	0.00	0.09

General Reference & Learning	0.13	-0.21
Instant Message	**0.28**	**-0.28**
Browsers	-0.03	-0.18
Video	-0.04	0.15
Writing	**0.25**	0.05
Search	-0.13	-0.21
Email	0.12	-0.21
General News & Opinion	-0.07	-0.10
Games	-0.06	-0.08
Presentation	**0.28**	0.17
General Shopping	-0.02	-0.01
Music	-0.08	-0.13

(Correlations in bold font are significant on a 5% level)

Regressions

Tables 6 and 7 show the results of the regression models that use subjective well-being (Table 6) and academic performance measures (Table 7) as the dependent variables. We use data from the two teaching blocks with “block 2” being a dummy variable indicating the block in which the treatment took place. Similarly, the “treatment group” refers to a dummy variable that identifies students that were exposed to the treatment. We use gender, age, number of years at the university, and whether the student is working next to the studies (dummy variable) as control variables.

Regarding subjective well-being measures SWLS and SWEMWBS, we do not see any significant effects due to using Facebook, Instagram, and Snapchat less. The (Treatment group * Block2 interaction) that indicates if the treatment group differed from the control group in block 2 remains insignificant, irrespectively of the well-being measure (SWLS or SWEMWBS) or sample used (full sample or Android subset). Only few predictors are significant (gender in the full sample, $p = 0.013$ or age in the Android subset, $p = 0.026$; both for the SWEMWBS) but these are not directly related to the experimental setting. Overall,

the amount of variance explained remains rather low and ranges between 3.2% (SWLS measure, full sample) and 14.2% (SWEMWBS, Android sample).

There is also no evidence that the treatment group achieves higher grades in block 2 (Treatment group * Block2 interaction), in which social media usage was restricted, than the control group ($p = 0.239$). This also holds for other subsets of the sample such as students with an Android device. Overall, the amount of variance explained in the grades remains very low with 3.4%. Only the number of years that the student has spent at the university is a significant positive predictor, i.e., Master's students achieve higher grades than Bachelor's.

In terms of number of credit points, i.e., number of courses attended, we do see a significant effect of restricting social media. While students overall attended courses with fewer credits in block 2 ($p = 0.004$) this is not the case for students in the treatment group such that they targeted significantly more credits ($p = 0.023$). With this dependent variable, the number of years at the university has a significant negative effect ($p = 0.002$) and, overall, 11.7% of variance in the number of credit points can be explained. The subset of Android users replicates these results, albeit generally with lower levels of significance due to the smaller sample size.

However, as noted above, the number of credit points does not necessarily show that students successfully passed more courses as also failed courses are included. Using the number of courses passed as the dependent variable shows that the students in the treatment group in fact do not differ from the control group (a Poisson model replicates these results). Thus, it appears that the treatment group attempted to pass more courses or courses with more credits compared to the control group but did not necessarily succeed.

Table 6: Regression of satisfaction with life and mental well-being measures (standardized coefficients in parentheses)

	Satisfaction with life (SWLS)				Mental well-being (SWEMWBS)			
	All users		Android		All users		Android	
	Estimate	p-value	Estimate	p-value	Estimate	p-value	Estimate	p-value
(Intercept)	**29.028**	**<0.001**	**36.967**	**<0.001**	**24.409**	**<0.001**	**30.497**	**<0.001**
Treatment group	-1.205 (-0.113)	0.221	-1.350 (-0.117)	0.380	-0.233 (-0.038)	0.676	-0.063 (-0.009)	0.942
Block 2	0.207 (0.019)	0.831	-0.552 (-0.048)	0.720	0.025 (0.004)	0.963	-0.342 (-0.050)	0.691
(Treatment group*Block2)	-0.253 (-0.020)	0.855	1.285 (0.097)	0.552	-0.126 (-0.018)	0.872	0.505 (0.065)	0.676
Gender (female)	-1.021 (-0.096)	0.143	-0.818 (-0.070)	0.455	**-0.991 (-0.162)**	**0.013**	-0.768 (-0.112)	0.210
Age in years	-0.161 (-0.096)	0.221	-0.494 (-0.217)	0.068	-0.057 (-0.059)	0.446	**-0.338 (-0.252)**	**0.026**
Years at the university	0.166 (0.048)	0.542	0.107 (0.028)	0.798	-0.139 (-0.069)	0.370	-0.089 (-0.040)	0.705
Working next to studies	0.280 (0.026)	0.692	-0.036 (-0.003)	0.975	0.724 (0.117)	0.072	1.020 (0.150)	0.117
R-squared	0.032		0.051		0.055		0.142	

Table 7: Regression of academic performance (standardized coefficients in parentheses)

	Regression of average grade				Regression of number of credit points				Regression of number of courses passed			
	All users		Android users		All users		Android users		All users		Android users	
	Estimate	p-value	Estimate	p-value	Estimate	p-value	Estimate	p-value	Estimate	p-value	Estimate	p-value
(Intercept)	**6.950**	**<0.001**	**6.578**	**<0.001**	**19.406**	**<0.001**	**13.393**	**0.009**	**3.904**	**<0.001**	**2.616**	**0.007**
Treatment group	0.237 (0.117)	0.205	0.536 (0.244)	0.067	-1.740 (-0.159)	0.071	-1.159 (-0.108)	0.388	-0.064 (-0.032)	0.721	0.124 (0.064)	0.625
Block 2	0.173 (0.086)	0.350	0.321 (0.146)	0.272	**-2.750 (-0.252)**	**0.004**	**-2.931 (-0.273)**	**0.031**	-0.347 (-0.172)	0.051	-0.345 (-0.177)	0.178
(Treatment group*Block2)	-0.310 (-0.132)	0.239	-0.592 (-0.235)	0.149	**3.090 (0.245)**	**0.023**	3.431 (0.278)	0.070	0.220 (0.094)	0.382	0.211 (0.094)	0.554
Gender (female)	0.142 (0.070)	0.281	0.137 (0.062)	0.507	0.706 (0.065)	0.300	0.388 (0.036)	0.684	0.201 (0.099)	0.113	0.090 (0.046)	0.618
Age in years	-0.016 (-0.048)	0.534	0.002 (0.005)	0.969	-0.130 (-0.076)	0.311	0.164 (0.078)	0.483	**-0.055 (-0.172)**	**0.022**	0.003 (0.007)	0.949
Years at the university	**0.114** (0.172)	**0.028**	0.083 (0.116)	0.294	**-0.854 (-0.239)**	**0.002**	**-1.232 (-0.352)**	**0.001**	**-0.109 (-0.165)**	**0.028**	-0.131 (-0.206)	0.060
Working next to studies	-0.043 (-0.021)	0.750	-0.202 (-0.092)	0.358	-0.009 (-0.001)	0.990	1.439 (0.134)	0.156	-0.082 (-0.040)	0.523	0.134 (0.069)	0.484
R-squared	0.034		0.054		0.117		0.159		0.113		0.083	

These null results of the effects of restricting social media usage on academic performance and subjective well-being raises the question of whether our study is underpowered to detect economically significant effects. At 80% power the minimum detectable difference in life satisfaction (on the SWLS scale) with our sample size is 2.5 on a scale of 5-35 (average life satisfaction score in our sample is 25 with a standard deviation of 5). The SWLS categories comprise 5 scale points, for example, a score of 25-29 is considered as a "high" score. Therefore, even if the treatment group's life satisfaction score increased by 1.5 it is generally not sufficient to change the classification from one category to another. The minimum detectable difference in average grade is 0.5 on a scale of 1-10 (average grade in our sample is about 7 with a standard deviation of 1). Given that students receive grades which are whole numbers, this threshold is still below the value that would increase the treatment group's average grade by a full point. At least, our results indicate that changes in grades and subjective well-being are not substantial so that they could be detected with our sample size. To further address potential concerns about statistical power we applied a hierarchical Bayes ANOVA (BANOVA) model that includes between and within subject effects and accommodates unobserved heterogeneity by including a normal distribution of the parameters across individuals [51]. All models converge and generally replicate the results above (details are available from the authors upon request).

Post-treatment Effects

The formal analysis of the post-treatment effects is based on a sample of 106 students who provided activity data throughout all three blocks and in all four surveys. While the treatment condition significantly reduced their social media usage in block 2 compared to the control group, this effect was not permanent. After we suspended the limit in block 3 the social media activities of users in the treatment group increased again, showing no significant differences to the control group any longer (t-test, $p = 0.668$). We further do not see any

significant differences between the treatment and control group in block 3 in terms of grades (t-test, $p = 0.152$), number of credit points (t-test, $p = 0.923$), satisfaction with life (t-test, $p = 0.499$), or mental well-being (t-test, $p = 0.966$), i.e., there is no lagged effect of reduced social media usage.

Discussion

In this paper, we analyzed the effects of restricting social media usage. We did not find significant causal effects of social media usage on well-being or academic performance, other than students attempting (but not succeeding) to pass more courses or courses with more credits. However, we found robust evidence of substitution effects that can potentially explain the null finding. Specifically, we showed that social media and instant messaging apps can be substitutes. The European Commission approved Facebook's acquisition of WhatsApp in 2014 based on Facebook's claim that it operates in a different market and does not compete directly with WhatsApp [52]. Our results indicate that they are in fact direct competitors. After acquiring WhatsApp, Facebook started automatically matching its users' profiles with their WhatsApp accounts and has started integrating WhatsApp, Instagram and Facebook user accounts [53]. The European Commission fined Facebook €110 million in 2017 for this practice because Facebook had provided misleading information about the feasibility of automatically matching profiles during its acquisition of WhatsApp. However while announcing this fine, the Commission still maintained its belief that Facebook and WhatsApp do not directly compete with each other [54]. Antitrust authorities should consider the market power of this combined entity if the world's biggest social media platforms are integrated with the world's biggest instant messaging platform. This finding also raises the question how to properly define social networks, for future academic studies or antitrust cases.

While we found null results estimating the causal impact of social media usage on well-being and academic performance, and not all null results matter, we believe that null results are interesting and important in this context. The media has hyped correlational studies showing a negative association between social media usage and well-being and it is important to balance this narrative through causal evidence. A limitation of our study is the lack of a larger sample size to detect smaller effects. While these small effects might not be economically significant, more research is needed using massive samples. Future research can also look at differences between Android and iOS users in more detail with larger samples since we lack power to analyze these differences in our current study. However, it is challenging to recruit a large number of subjects from a representative sample for a long-term study. Direct collaborations with social media platforms or internet service providers (which control internet traffic) could be a way of obtaining data from larger samples. These would also facilitate more targeted interventions such as restricting only content consumption or social interactions on social media [18].

It is interesting to notice that while social media generates large amount of consumer surplus [3], it doesn’t seem to affect the subjective well-being of users. Future research can explore this wedge between consumer surplus and subjective well-being and see whether they are correlated for some products and uncorrelated for others. Future research should also explore the addictiveness of social media in more detail [55]. Our findings in block 3 show that the students in the treatment condition go back to their old habits and do not adopt a lower social media usage that they experienced in block 2. On the other hand, showing students how much time they are spending on social networks via the software seems to have an overall negative trend on its usage (comparing usage in block 1 and block 3). Curing social media addiction (if it is indeed addiction) might therefore be a longer process. Future research can look at mechanisms for the emergence of social media addiction, for e.g. through targeted

advertising or news feed algorithms and features of social media apps (e.g. video sharing) which are correlated with addiction.

Moreover, due to our student sample implications for the general population are limited. It could be that students use social media mostly for communication purposes and therefore show significant substitution effects with instant messaging. We might see different effects for users who visit social media for content consumption, e.g., watching videos, instead of social interaction [18]. In this regard, newer social media services such as TikTok might have a different effect. However, these and other comparable social media services were not relevant at the time we conducted the study (as we measured in block 1). More research is needed looking at emerging social media platforms such as TikTok. Moreover, one could define social media more broadly to include instant messaging services and implement a stricter restriction involving all types of communication and social networking apps. However, conducting such a study for any length of time is a challenging endeavor. Additionally, while we study the impact of social media on students and academic performance, future research can look at workplace settings and study the impact of social media and its substitutes on worker productivity and well-being. We believe that rigorous causal evidence through randomized controlled trials and objectively measured time spent is the way forward in addressing questions regarding the impact of technology on well-being. The widespread adoption of most major technologies in the past such as radio, television, video games and computers was followed with unfounded fears about their impact on well-being. This story repeats again with social media. We find that social media usage does not cause lower well-being or poor academic performance. Rather, we demonstrate that students find other means of social networking using instant messaging when exogenously restricting their social media usage. To conclude: You can take social networking away from the students, but you cannot take students away from their social network.

References

1. Statista. Most popular social networks worldwide as of January 2022, ranked by number of monthly active users. Jan 2022 [Cited 2022 February 18]. Available from: https://www.statista.com/statistics/272014/global-social-networks-ranked-by-number-of-users/
2. Nielsen. Time Flies: U.S. Adults Now Spend Nearly Half a Day Interacting with Media. 2018 July 31 [Cited 2019 May 6]. Available from: https://www.nielsen.com/us/en/insights/news/2018/time-flies-us-adults-now-spend-nearly-half-a-day-interacting-with-media.html
3. Brynjolfsson E, Collis A, Eggers F. Using massive online choice experiments to measure changes in well-being. Proceedings of the National Academy of Sciences. 2019 Apr 9;116(15):7250-5.
4. Haidt J. Social Media Use and Mental Health: A Review. 2019 [Cited 2019 May 6]. Available from: https://docs.google.com/document/d/1w-HOfseF2wF9YIpXwUUtP65-olnkPyWcgF5BiAtBEy0/
5. Cheng J, Burke M, Davis EG. Understanding perceptions of problematic Facebook use: When people experience negative life impact and a lack of control. InProceedings of the 2019 CHI conference on human factors in computing systems 2019 May 2 (pp. 1-13).
6. Orben A, Dienlin T, Przybylski AK. Social media's enduring effect on adolescent life satisfaction. Proceedings of the National Academy of Sciences. 2019 May 21;116(21):10226-8.
7. Rubin AJ, Peltier E. France Bans Smartphones in Schools Through 9th Grade. Will It Help Students?. The New York Times. 2018 September 20 [Cited 2019 May 11].

Available from: https://www.nytimes.com/2018/09/20/world/europe/france-smartphones-schools.html

8. American Academy of Pediatrics. American Academy of Pediatrics Announces New Recommendations for Childrens Media Use. 2019 [Cited 2019 May 11]. Available from: https://www.aap.org/en-us/about-the-aap/aap-press-room/Pages/American-Academy-of-Pediatrics-Announces-New-Recommendations-for-Childrens-Media-Use.aspx
9. Orben A, Przybylski AK. The association between adolescent well-being and digital technology use. Nature human behaviour. 2019 Feb;3(2):173-82.
10. Kraut R, Burke M. Internet use and psychological well-being: Effects of activity and audience. Communications of the ACM. 2015 Nov 23;58(12):94-100.
11. Shakya HB, Christakis NA. Association of Facebook use with compromised well-being: A longitudinal study. American journal of epidemiology. 2017 Feb 1;185(3):203-11.
12. Burke M, Marlow C, Lento T. Social network activity and social well-being. InProceedings of the SIGCHI conference on human factors in computing systems 2010 Apr 10 (pp. 1909-1912).
13. Hobbs WR, Burke M, Christakis NA, Fowler JH. Online social integration is associated with reduced mortality risk. Proceedings of the National Academy of Sciences. 2016 Nov 15;113(46):12980-4.
14. Burke M, Kraut RE. The relationship between Facebook use and well-being depends on communication type and tie strength. Journal of computer-mediated communication. 2016 Jul 1;21(4):265-81.

15. Kim C, Shen C. Connecting activities on Social Network Sites and life satisfaction: A comparison of older and younger users. Computers in Human Behavior. 2020 Apr 1;105:106222.
16. Schemer C, Masur PK, Geiß S, Müller P, Schäfer S. The impact of internet and social media use on well-being: A longitudinal analysis of adolescents across nine years. Journal of Computer-Mediated Communication. 2021 Jan;26(1):1-21.
17. Johannes N, Meier A, Reinecke L, Ehlert S, Setiawan DN, Walasek N, Dienlin T, Buijzen M, Veling H. The relationship between online vigilance and affective well-being in everyday life: Combining smartphone logging with experience sampling. Media Psychology. 2021 Sep 3;24(5):581-605.
18. Liu D, Baumeister RF, Yang CC, Hu B. Digital communication media use and psychological well-being: A meta-analysis. Journal of Computer-Mediated Communication. 2019 Sep;24(5):259-73.
19. Huang C. Time spent on social network sites and psychological well-being: A meta-analysis. Cyberpsychology, Behavior, and Social Networking. 2017 Jun 1;20(6):346-54.
20. Appel M, Marker C, Gnambs T. Are social media ruining our lives? A review of meta-analytic evidence. Review of General Psychology. 2020 Mar;24(1):60-74.
21. Meier A, Reinecke L. Computer-mediated communication, social media, and mental health: A conceptual and empirical meta-review. Communication Research. 2021 Dec;48(8):1182-209.
22. Dienlin T, Johannes N. The impact of digital technology use on adolescent well-being. Dialogues in clinical neuroscience. 2022 Apr 1.
23. Masur PK. Digital communication effects on loneliness and life satisfaction. InOxford Research Encyclopedia of Communication 2021 Jun 28.

24. Orben A. Teenagers, screens and social media: a narrative review of reviews and key studies. Social psychiatry and psychiatric epidemiology. 2020 Apr;55(4):407-14.
25. Sewall CJ, Goldstein TR, Wright AG, Rosen D. Does Objectively Measured Social-Media or Smartphone Use Predict Depression, Anxiety, or Social Isolation Among Young Adults?. Clinical Psychological Science. 2022 Mar 25:21677026221078309.
26. Junco R. Comparing actual and self-reported measures of Facebook use. Computers in Human Behavior. 2013 May 1;29(3):626-31.
27. Scharkow M. The accuracy of self-reported internet use—A validation study using client log data. Communication Methods and Measures. 2016 Jan 2;10(1):13-27.
28. Ellis DA, Davidson BI, Shaw H, Geyer K. Do smartphone usage scales predict behavior?. International Journal of Human-Computer Studies. 2019 Oct 1;130:86-92.
29. Ellis DA. Are smartphones really that bad? Improving the psychological measurement of technology-related behaviors. Computers in Human Behavior. 2019 Aug 1;97:60-6.
30. Verduyn P, Lee DS, Park J, Shablack H, Orvell A, Bayer J, Ybarra O, Jonides J, Kross E. Passive Facebook usage undermines affective well-being: Experimental and longitudinal evidence. Journal of Experimental Psychology: General. 2015 Apr;144(2):480.
31. Marotta V, Acquisti A. Interrupting interruptions: A digital experiment on social media and performance. Available at SSRN 3283951. 2018 Aug 13.
32. Carter SP, Greenberg K, Walker MS. The impact of computer usage on academic performance: Evidence from a randomized trial at the United States Military Academy. Economics of Education Review. 2017 Feb 1;56:118-32.
33. Belo R, Ferreira P, Telang R. Broadband in school: Impact on student performance. Management Science. 2014 Feb;60(2):265-82.

34. Beland LP, Murphy R. Ill communication: technology, distraction & student performance. Labour Economics. 2016 Aug 1;41:61-76.

35. Allcott H, Braghieri L, Eichmeyer S, Gentzkow M. The welfare effects of social media. American Economic Review. 2020 Mar;110(3):629-76.

36. Brailovskaia J, Ströse F, Schillack H, Margraf J. Less Facebook use–More well-being and a healthier lifestyle? An experimental intervention study. Computers in Human Behavior. 2020 Jul 1;108:106332.

37. Hall JA, Xing C, Ross EM, Johnson RM. Experimentally manipulating social media abstinence: results of a four-week diary study. Media Psychology. 2021 Mar 4;24(2):259-75.

38. Hinsch C, Sheldon KM. The impact of frequent social Internet consumption: Increased procrastination and lower life satisfaction. Journal of Consumer Behaviour. 2013 Nov;12(6):496-505.

39. Tromholt M. The Facebook experiment: Quitting Facebook leads to higher levels of well-being. Cyberpsychology, behavior, and social networking. 2016 Nov 1;19(11):661-6.

40. Mosquera R, Odunowo M, McNamara T, Guo X, Petrie R. The economic effects of Facebook. Experimental Economics. 2020 Jun;23(2):575-602.

41. Hanley SM, Watt SE, Coventry W. Taking a break: The effect of taking a vacation from Facebook and Instagram on subjective well-being. Plos one. 2019 Jun 6;14(6):e0217743.

42. Vanman EJ, Baker R, Tobin SJ. The burden of online friends: The effects of giving up Facebook on stress and well-being. The Journal of social psychology. 2018 Jul 4;158(4):496-508.

43. Przybylski AK, Weinstein N. A large-scale test of the goldilocks hypothesis: quantifying the relations between digital-screen use and the mental well-being of adolescents. Psychological science. 2017 Feb;28(2):204-15.

44. Diener ED, Emmons RA, Larsen RJ, Griffin S. The satisfaction with life scale. Journal of personality assessment. 1985 Feb 1;49(1):71-5.

45. Kross E, Verduyn P, Demiralp E, Park J, Lee DS, Lin N, Shablack H, Jonides J, Ybarra O. Facebook use predicts declines in subjective well-being in young adults. PloS one. 2013 Aug 14;8(8):e69841.

46. Haerpfer C, Inglehart R, Moreno A, Welzel C, Kizilova K, Diez-Medrano J. M. Lagos, P. Norris, E. Ponarin & B. Puranen et al.(Eds.).(2020). World Values Survey: Round Seven-Country-Pooled Datafile. 2020.

47. Kahneman D, Deaton A. High income improves evaluation of life but not emotional well-being. Proceedings of the national academy of sciences. 2010 Sep 21;107(38):16489-93.

48. Perez-Truglia R. The effects of income transparency on well-being: Evidence from a natural experiment. American Economic Review. 2020 Apr;110(4):1019-54.

49. Tennant R, Hiller L, Fishwick R, Platt S, Joseph S, Weich S, Parkinson J, Secker J, Stewart-Brown S. The Warwick-Edinburgh mental well-being scale (WEMWBS): development and UK validation. Health and Quality of life Outcomes. 2007 Dec;5(1):1-3.

50. Stewart-Brown S, Tennant A, Tennant R, Platt S, Parkinson J, Weich S. Internal construct validity of the Warwick-Edinburgh mental well-being scale (WEMWBS): a Rasch analysis using data from the Scottish health education population survey. Health and quality of life outcomes. 2009 Dec;7(1):1-8.

51. Wedel M, Dong C. Banova: Bayesian analysis of experiments in consumer psychology. Journal of Consumer Psychology. 2020 Jan;30(1):3-23.

52. European Commission. Mergers: Commission approves acquisition of WhatsApp by Facebook. 2014 October 3 [Cited 2019 June 1]. Available from: http://europa.eu/rapid/press-release_IP-14-1088_en.htm

53. Isaac M. Zuckerberg Plans to Integrate WhatsApp, Instagram and Facebook Messenger. 2019 January 25 [Cited 2019 June 1]. Available from: https://www.nytimes.com/2019/01/25/technology/facebook-instagram-whatsapp-messenger.html

54. European Commission. Mergers: Commission fines Facebook €110 million for providing misleading information about WhatsApp takeover. 2017 May 18 [Cited 2019 June 1]. Available from: http://europa.eu/rapid/press-release_IP-17-1369_en.htm

55. Allcott H, Gentzkow M, Song L. Digital addiction. National Bureau of Economic Research; 2021 Jun 21.

Figures

Figure 1: All digital activities and social media usage over time (users with Android devices)

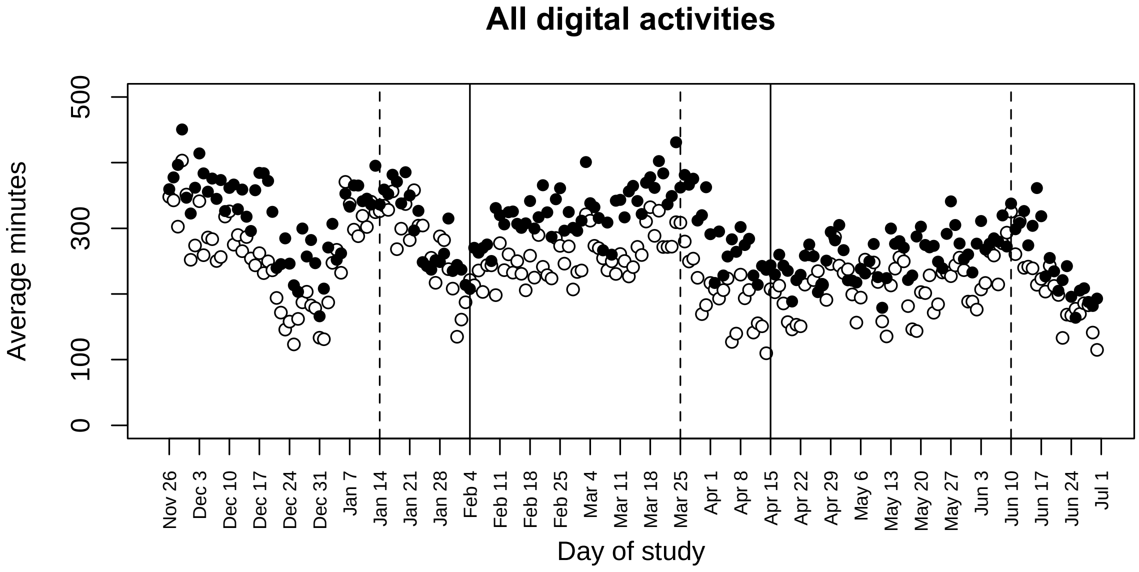

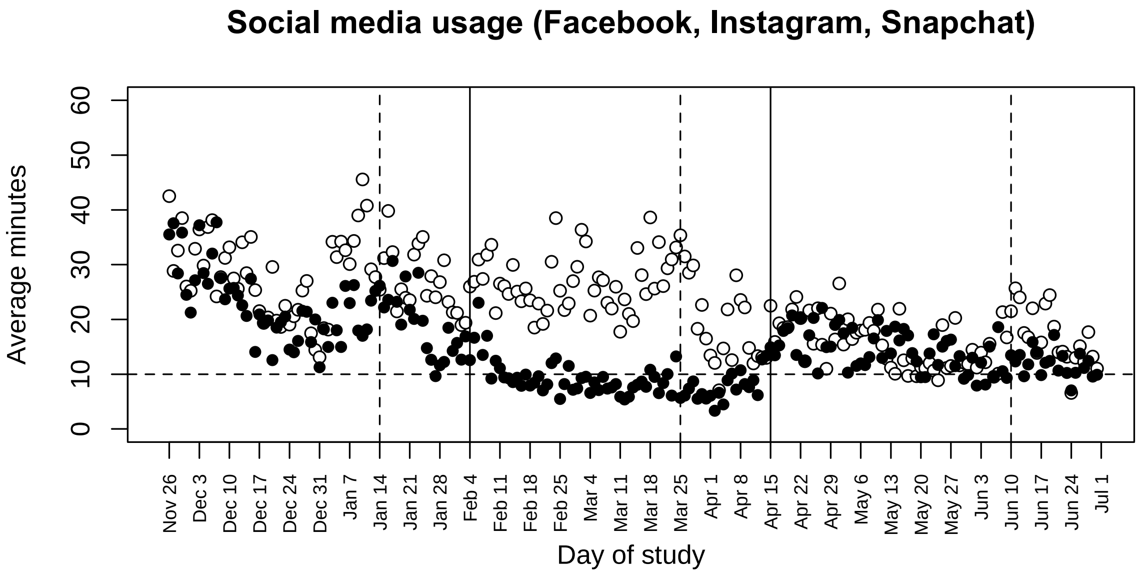

black = treatment, white = control group
solid vertical lines: start of a new teaching block
dashed vertical lines: start of the exam period

Figure 2.1: Tracked digital activities over time (users with Android devices)

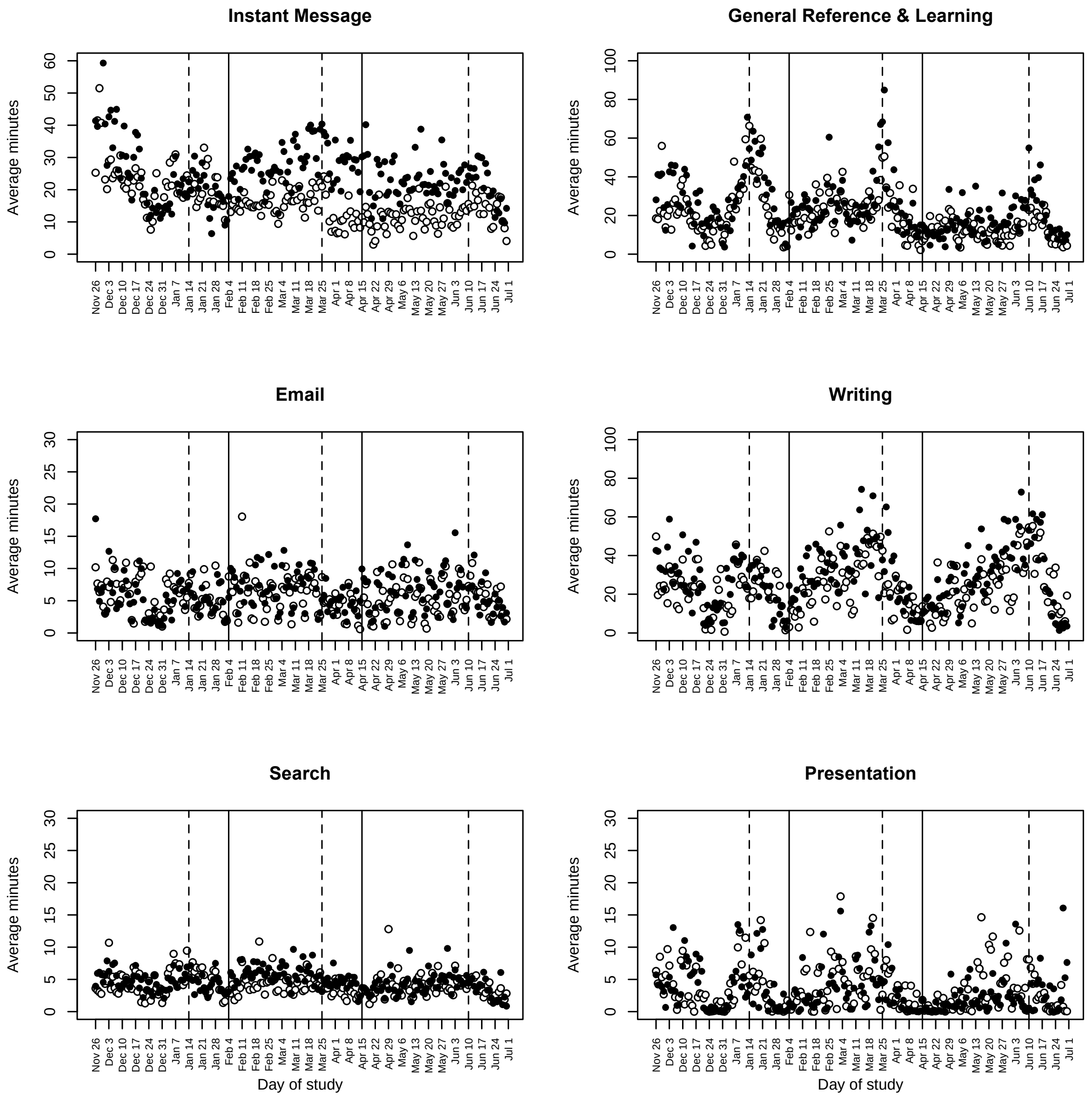

black = treatment, white = control group
solid vertical lines: start of a new teaching block
dashed vertical lines: start of the exam period

Figure 2.2: Tracked digital activities over time (users with Android devices)

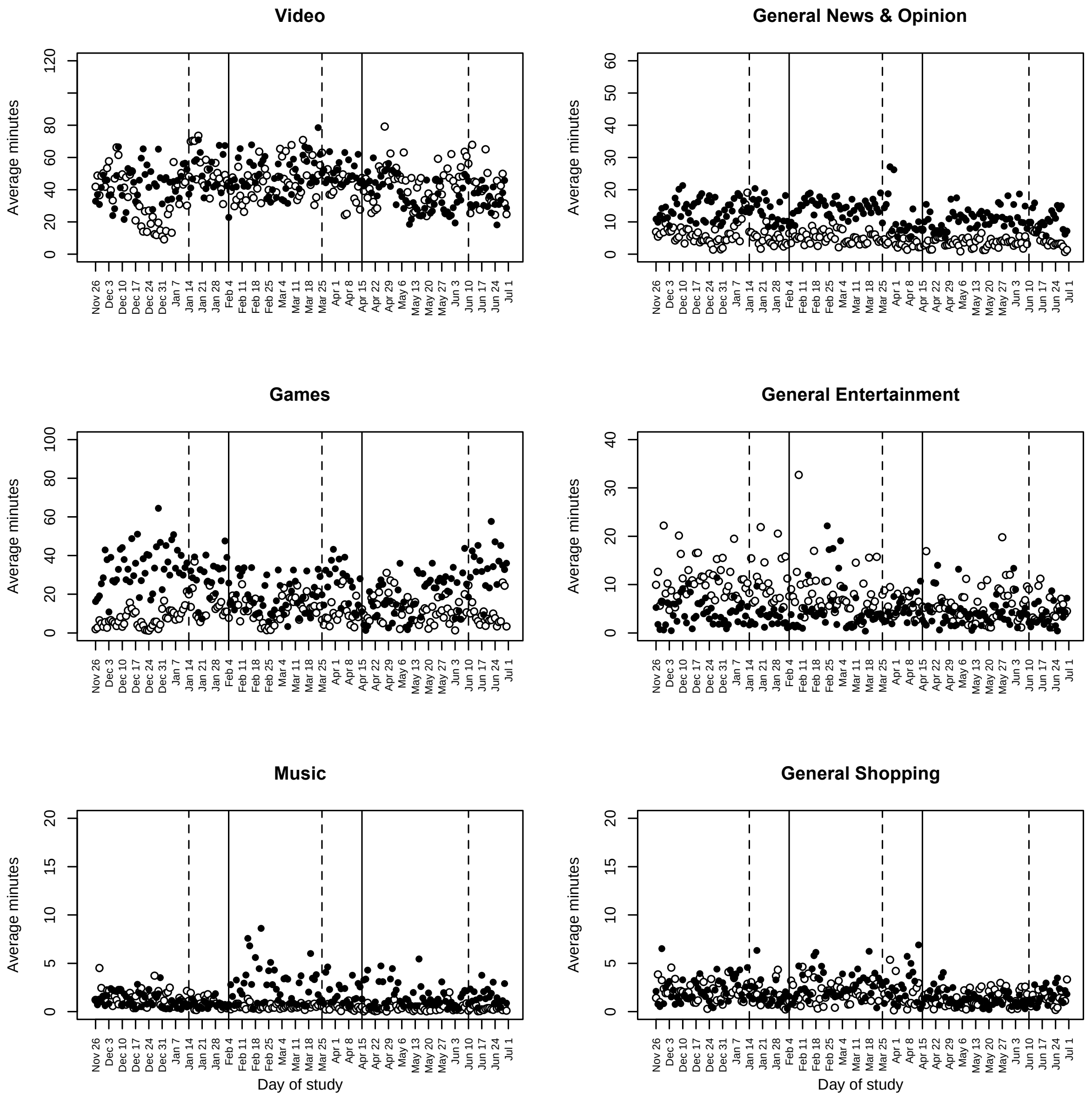

black = treatment, white = control group
solid vertical lines: start of a new teaching block
dashed vertical lines: start of the exam period

Figure 3: Differences in subjective well-being measures

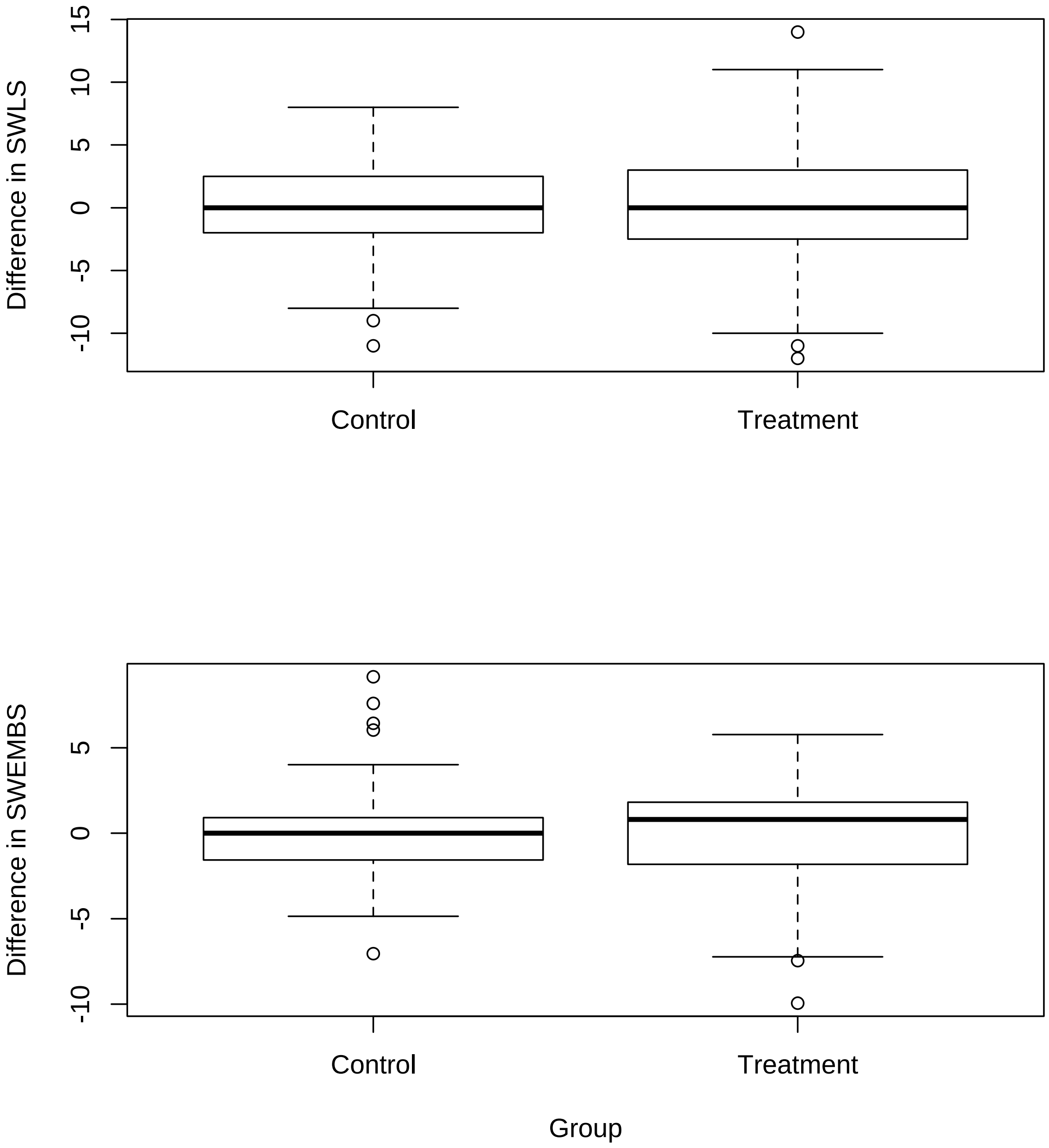

Figure 4: Differences in academic performance

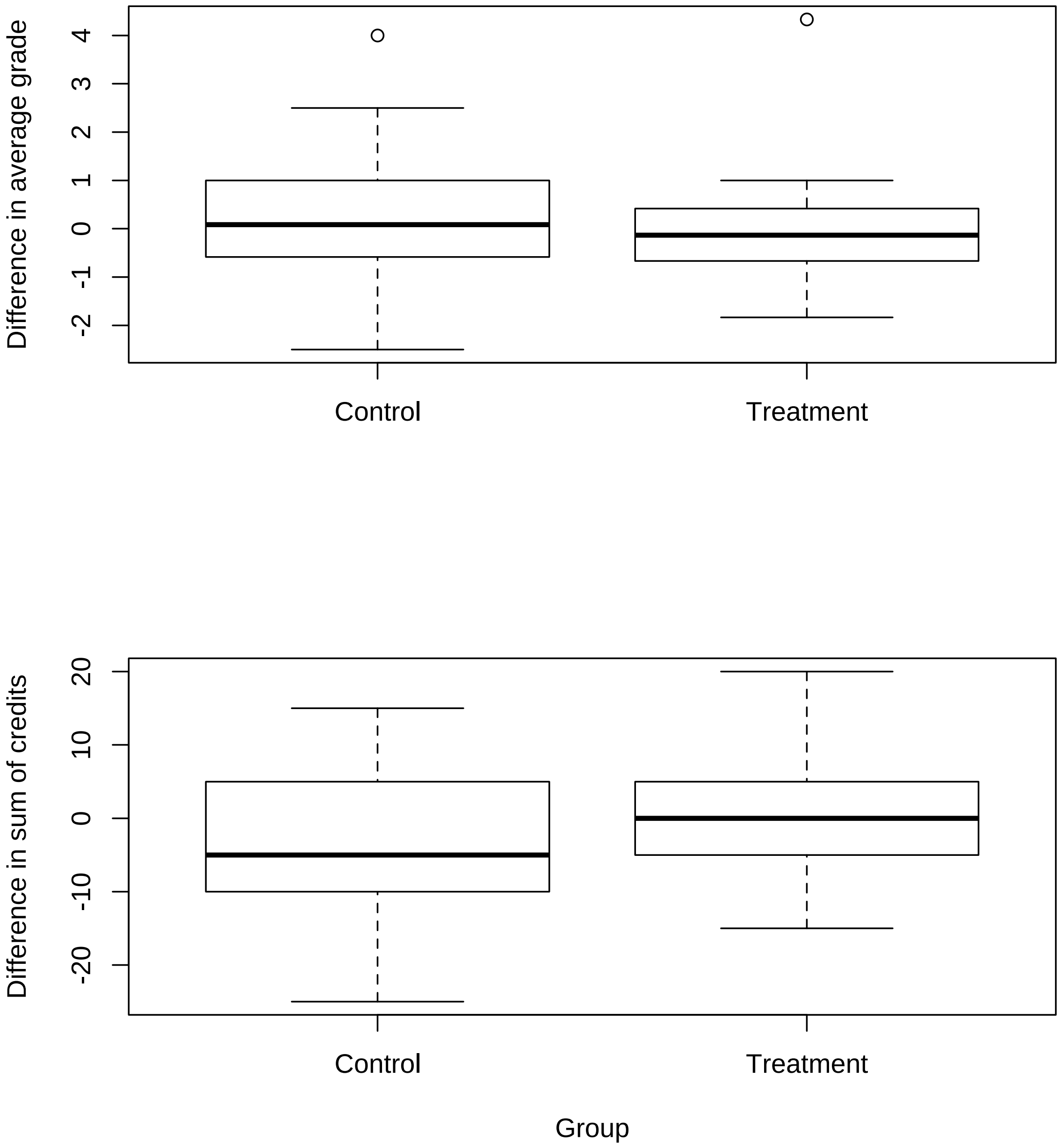

Supporting Information

Figure S1: All digital activities and social media usage over time (all users)

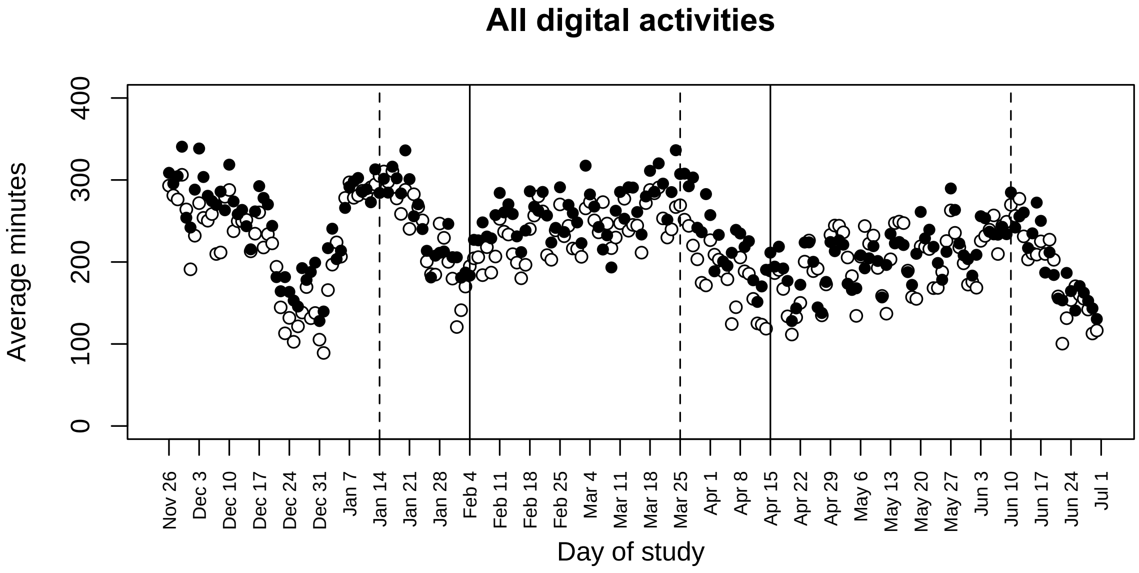

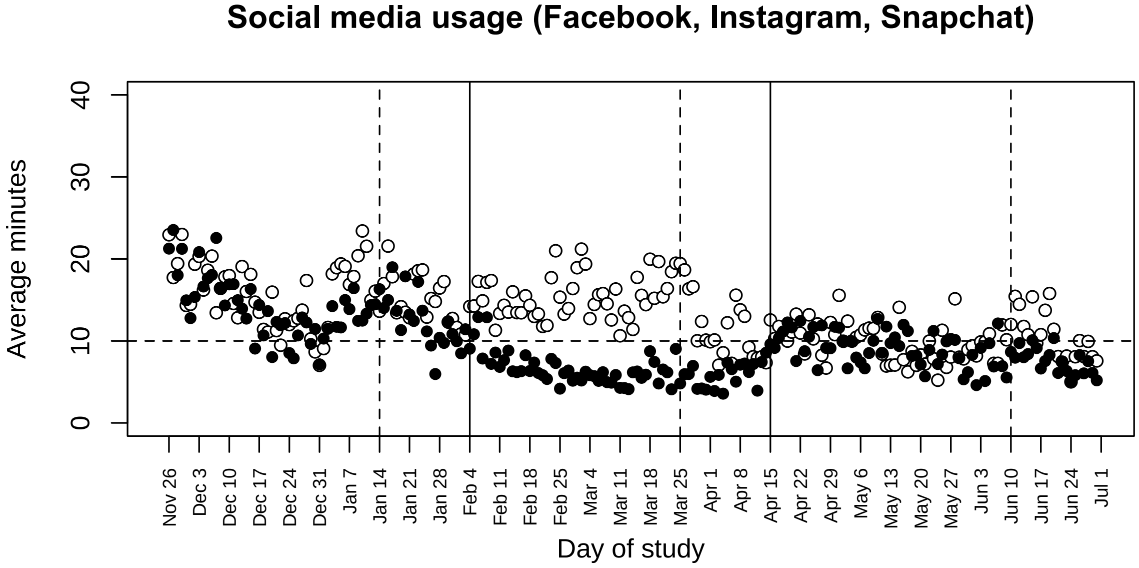

black = treatment, white = control group
solid vertical lines: start of a new teaching block
dashed vertical lines: start of the exam period

Figure S2.1: Tracked digital activities over time (all users)

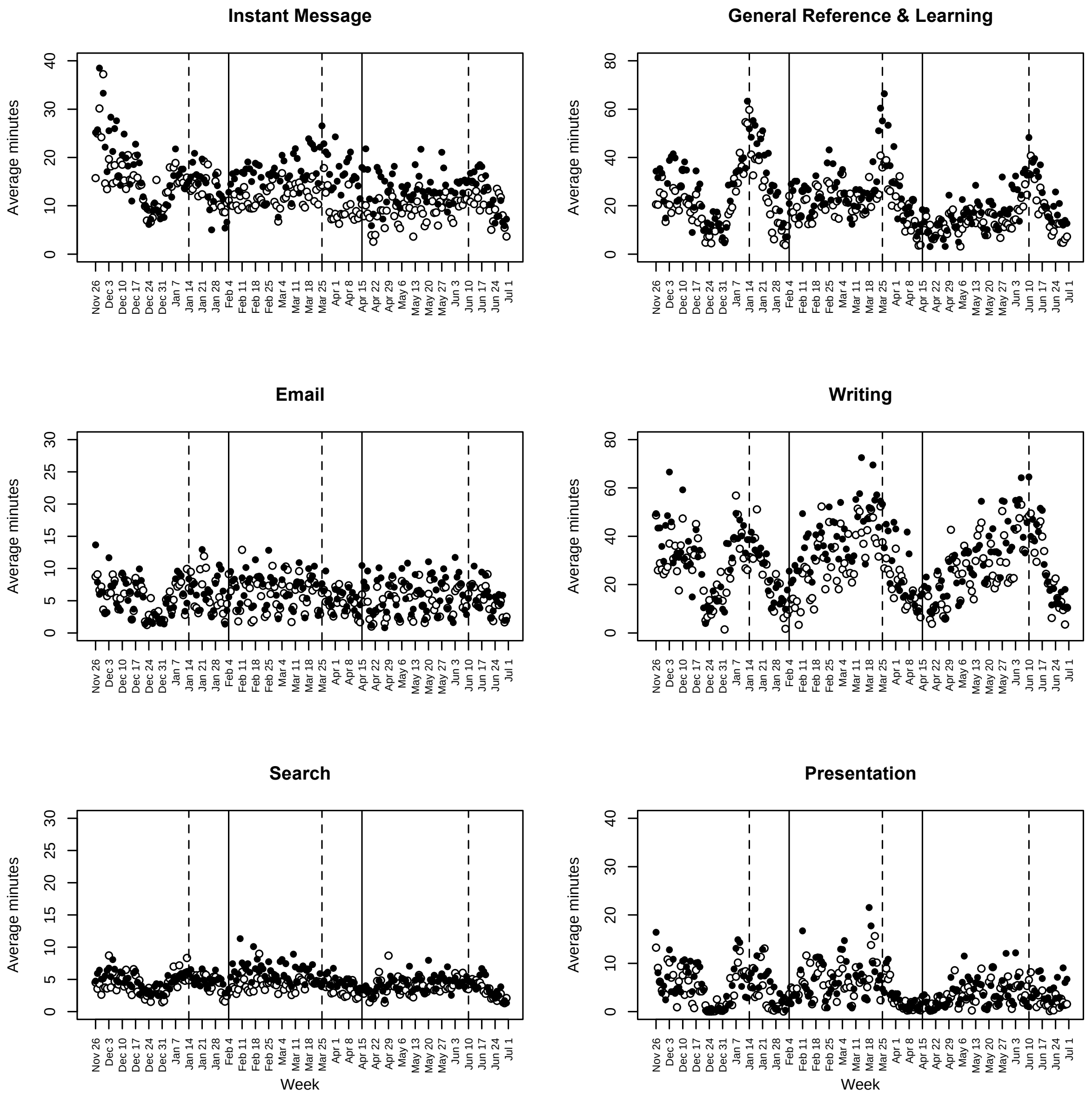

black = treatment, white = control group
solid vertical lines: start of a new teaching block
dashed vertical lines: start of the exam period

Figure S2.2: Tracked digital activities over time (all users)

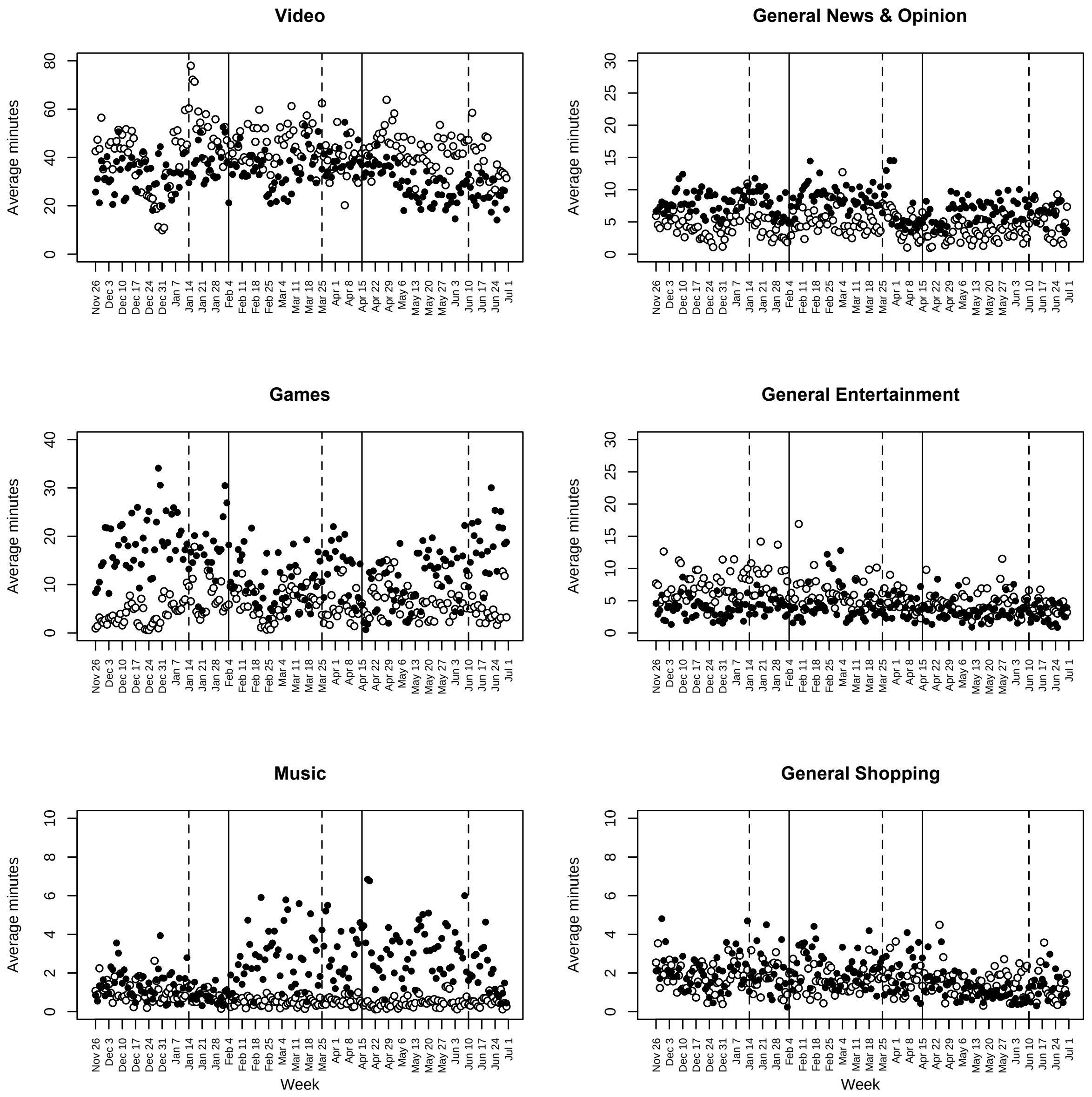

black = treatment, white = control group
solid vertical lines: start of a new teaching block
dashed vertical lines: start of the exam period

Table S1: Significance of differences between treatment and control group in block 2 vs. block 1 (difference-in-differences)

Category	p-value all users	p-value Android users
Social media	**<0.001**	**0.013**
General Reference & Learning	0.811	0.491
Instant Message	0.123	**0.008**
Browsers	0.628	0.257
Video	0.513	0.715
Writing	0.532	0.304
Search	0.745	0.494
Email	0.352	0.104
News & Opinion	0.864	0.543
General Entertainment	0.679	0.506
Games	0.411	0.662
Presentation	0.857	0.803
General Shopping	0.833	0.611
Music	0.099	**0.027**

All variables measured on a log scale.

Table S2: Summary statistics for well-being measures

Measure	Full sample		Treatment group		Control group	
	Mean	SD	Mean	SD	Mean	SD
SWLS survey 1	25.0	5.5	24.3	5.9	25.7	5.1
SWLS survey 2	25.0	5.4	24.4	5.4	25.6	5.3
SWLS survey 3	25.1	5.3	24.4	5.3	25.7	5.3
SWEMWBS survey 1	22.8	3.0	23.0	2.8	22.5	3.1
SWEMWBS survey 2	22.5	2.7	22.4	2.6	22.5	2.9
SWEMWBS survey 3	22.4	3.3	22.4	3.6	22.5	3.1

Revised Manuscript with Track Changes

Effects of restricting social media usage on wellbeing and performance: A randomized control trial among students

Avinash Collis[1*37¶], Felix Eggers[2*¶]

[1] McCombs School of Business, The University of Texas at Austin, AusinAustin, Texas, USA

[2] Copenhagen Business School, Frederiksberg, Copenhagen, Denmark

*Corresponding authors

Email: avinash.collis@mccombs.utexas.edu (AC), fe.marktg@cbs.dk (FE)

¶These authors contributed equally to this work.

Formatted: German (Germany)

Abstract

Recent research has shown that social media services create large consumer surplus. Despite their positive impact on economic welfare, concerns are raised about the negative association between social media usage and ~~performance or~~ well-being or performance. However, causal empirical evidence is still scarce. To address this research gap, we conduct a randomized controlled trial among students in which we track participants' daily digital activities over the course of three quarters of an academic year. In the experiment, we randomly allocate half of the sample to a treatment condition in which social media usage (Facebook, Instagram, and Snapchat) is restricted to a maximum of 10 minutes per day. We find that participants in the treatment group substitute social media for instant messaging and do not decrease their total time spent on digital devices. Contrary to findings from previous correlational studies, we do not find any significant impact of social media usage as it was defined in our study on well-being and academic success. Our results also suggest that antitrust authorities should consider instant messaging and social media services as direct competitors before approving acquisitions.

Introduction

Social media increasingly plays an important role in our daily lives. Ever since the launch of major modern social media platforms such as Facebook, users have adopted them at an explosive pace and adoption continues to increase to this day. Almost 3 billion users are active monthly on Facebook in 2021[1]. This corresponds to over a third of the global population. The average adult spends over 45 minutes every day on social media platforms[2].

[1] Source: Statista (https://www.statista.com/statistics/272014/global-social-networks-ranked-by-number-of-users/), accessed on February 18, 2022)
[2] Source: Nielsen (https://www.nielsen.com/us/en/insights/news/2018/time-flies-us-adults-now-spend-nearly-half-a-day-interacting-with-media.html, accessed on May 6, 2019)

Given this rapid adoption and usage of social media platforms, it is essential to study the impact of social media on the well-being of users. Brynjolfsson, Collis and Eggers (2019) find that digital technologies, including social media, generate a large amount of consumer surplus. More specifically, they conduct incentive compatible choice experiments to measure the consumer surplus generated by Facebook and find that the median US Facebook user obtains around $48/month of value from using Facebook in 2017 as measured from their willingness to accept to give up access to Facebook for a month. They also conduct a similar experiment with students at a large European university and find that the median student in their sample obtains €97/month of value from using Facebook.

While Facebook and other social media services seem to generate a large amount of consumer surplus and contribute towards the economic well-being of their users, questions are raised about the negative externalities generated by social media. There is an active debate in media and academic research about the impact of social media on subjective well-being (including happiness and life satisfaction) and productivity. Current empirical results are ambiguous. Across different studies, correlational evidence points towards a positive, neutral (null results) and negative relationship between social media use and well-being (see Haidt (2019) for a comprehensive literature review of social media use and mental health). However, most of this evidence suffers from issues related to reverse causality (Cheng, Burke and Davis (2019)) and inaccurate measures of self-reported social media use (Orben, Dienlin and Przybylski (2019)). Rigorous causal evidence on long term impacts of social media use on well-being is lacking.

Concerns are also raised in the field of Education policy on the impact of screen time (including social media use) on academic performance of students. Critics contend that social media use on smartphones distracts students from focusing in classes and affects their grades. Motivated by these concerns, the French education ministry banned smartphones in schools

from first through ninth grades[3]. The American Academy of Pediatrics also recommends parents to limit the time spent by children and adolescents on social media so that they have enough time left to study[4]. However, a rigorous analysis of the data used in previous correlational studies that were used as evidence to support these policies suggests that the effects of social media use and screen time on adolescent well-being are too small to warrant policy changes (Orben and Przybylski (2019)).

Given these widespread concerns and conflicting correlational evidence on the impact of social media on well-being, it is necessary to obtain causal evidence in a timely manner before policies are implemented hastily. We seek to fill this research gap by conducting a randomized controlled trial to measure the causal long-term impact of social media use on academic performance and well-being. Specifically, the research question we aim to answer is: How does restricting social media use affect a) well-being, b) academic performance, and c) other digital activities?

We recruit students at a large European university to be part of our study over the course of three academic terms (quarters). The subjects install a software (RescueTime) on their personal computers and mobile devices. This software tracks all of the digital activities of the subjects during the entire duration of the study period. The first term serves as the baseline period. In the second term, subjects are randomized into treatment and control groups and the treatment group has social media use (Facebook, Instagram and Snapchat) restricted to a maximum of 10 minutes per day across all devices. We then measure the post-treatment effects in the third term.

---

[3] Source: The New York Times (https://www.nytimes.com/2018/09/20/world/europe/france-smartphones-schools.html, accessed on May 11, 2019)

[4] Source: American Academy of Pediatrics (https://www.aap.org/en-us/about-the-aap/aap-press-room/Pages/American-Academy-of-Pediatrics-Announces-New-Recommendations-for-Childrens-Media-Use.aspx, accessed on May 11, 2019)

We observe the entire space of digital activities performed by our subjects that covers online and also offline activities on their devices, including activities related to learning (such as writing in Microsoft Word or reading a PDF). Our social media use metrics are computed based on the actual time spent on social media and are not based on self-reported metrics of time spent, which is predominantly used in the existing literature. In addition to the digital activities, we obtain objective metrics of performance (grades) and subjective well-being scores solicited through surveys.

Contrary to results from previous studies using observational data, we do not find evidence that social media causes a positive or negative impact on well-being (including life satisfaction and mental health). Moreover, we also do not find any evidence that social media usage impacts academic success. However, we find significant substitution effects. Specifically, we see that participants in the treatment group substituted their use of social media services for instant messaging apps (e.g. WhatsApp). In total, these participants do not spend less time on their digital devices (computers and mobile phones) as those in the control group.

Our paper makes three main contributions. First, we test the popular media narrative portraying social media as the villain responsible for negatively affecting well-being of society. We do not find any evidence supporting this hypothesis. Second, educators and parents are increasingly concerned about the impact of digital distractions on academic performance and are restricting the online activities of students (for example through parental control software or by taking away their devices). While previous evidence seems to suggest that device usage in class might negatively affect academic performance, our results show that restricting social media usage from the lives of students (inside and outside class) might not have the intended effect. Finally, our paper is one of the first to provide evidence of substitutability between social media and instant messaging apps. This has major

implications for antitrust authorities analyzing the market power of major social media platforms such as Facebook which owns Instagram (another social media service) and WhatsApp (instant messaging service).

The paper proceeds as follows. In the next section, we provide a brief review of existing literature on the impact of social media use on well-being and academic performance. In the following section, we describe the design of our experiment and data collected over the course of the study. We then show the main results and conclude with a discussion of the limitations of this study and directions for future research.

Related Literature

The impact of the internet in general, and social media in particular, on well-being has attracted the attention of a number of researchers in the fields of psychology, epidemiology and human-computer interaction (HCI) over the past two decades. Most of this literature uses self-reported metrics of technology use and provides cross-sectional correlational evidence. Kraut and Burke (2015) provide a review of this literature and express skepticism regarding cross-sectional and survey-based studies due to the presence of several confounding factors. Moreover, correlational studies might suffer from an abundance of researcher degrees of freedom and the file drawer problem such that only significant results are published, inevitably leading to the implication that social media usage either has a positive or negative effect. However, a null result or insignificant findings regarding social media usage might be a plausible outcome.

Orben and Przybylski (2019) rigorously analyze popular large scale social datasets (n=350k) used in previous correlational studies studying the impact of technology use on well-being by conducting a specification curve analysis of the data. This analysis involves running all possible analytical models using various combinations of the covariates. Instead of selective reporting, results from all of these analyses are reported. They find a small negative

association between digital technology use and adolescent well-being. However this effect is economically insignificant explaining at most 0.4% of the variation in well-being. For comparison, the authors show that seemingly neutral activities such as eating potatoes have the same negative association with well-being as technology use. Given these concerns with correlational analyses involving cross sectional data, Kraut and Burke (2015) call for experimental evidence paired with tracking data to provide reliable evidence on the relationship between internet use and well-being.

The subset of literature focusing on the association between social media use and well-being has found a wide range of effects (negative, mixed, positive and null). Using a longitudinal survey, Shakya and Christakis (2017) found a negative association between Facebook use and well-being. In contrast, Burke, Marlow and Lento (2010) find a positive association between directed communication on Facebook and social well-being due to subjects reporting improved feelings of social bonding and reduced loneliness. Similarly, Hobbs et al. (2016) match Facebook profiles with public health records and find that being more socially integrated online (by accepting more Facebook friends) is associated with reduced risk of mortality. Burke and Kraut (2016) find that targeted messages from strong ties is associated with positive improvements in well-being while viewing messages from friends broadcasted to all of their friends and receiving one-click feedbacks were not associated with any improvement in well-being. Kim and Shen (2020) find that directed communication activities on social media platforms are positively associated with life satisfaction for older users. Schemer et al. (2021) and Johannes et al. (2020) do not find any substantial relationship between social media use and subjective well-being. Liu et al. (2019) conduct a meta-analysis of 124 studies and find that the association between social media use and well-being depends on the type of social media use. This association is positive for social interactions and negative for content consumption. Huang (2017) also conducted a meta-analysis of 61 studies

and documented a weak correlation between time spent on social media and negative indicators including depression and loneliness.[5] Cheng, Burke and Davis (2019) combine a survey of Facebook users with their Facebook activities and find that subjects reporting problematic use of Facebook were also going through a major life event such as a breakup. This shows that confounding variables could be a major concern in previous studies associating social media use and well-being.

Orben, Dienlin and Przybylski (2019) use a large-scale longitudinal dataset and conduct a specification curve analysis to rigorously analyze the relationship between adolescent social media use and well-being. Most of the analyses report tiny, trivial and insignificant results. Moreover, they provide evidence for reverse causality showing that social media use predicts well-being in the future and vice versa. Similarly, Sewall et al. (2022) also find little to no evidence that changes in social media or smart phone use predict psychological distress.

Another major concern related to existing studies is the use of self-reported usage data. Survey respondents are typically asked to report the average time they spend on the internet, social media and digital devices. Several papers show that self-reported measures of technology use (including social media usage) are poorly correlated with actual usage and contain systematic patterns of misreporting (Junco 2013, Scharkow 2016, Ellis et al. 2019, Ellis 2019).

Given this inconclusive evidence and lack of objective technology use data in existing literature, it is essential to obtain reliable causal evidence in a timely manner to inform policy makers. We aim to resolve this gap by obtaining evidence through a randomized controlled trial and using objective technology use metrics tracked by a software installed on the digital devices of our experimental subjects. In terms of outcome variables, we track measures of

[5] For a comprehensive list of all studies studying social media use and well-being, see literature reviews by Appel et al. (2020), Meier and Reinecke (2021), Dienlin and Johannes (2020), Masur (2021) and Orben (2020).

subjective well-being (life satisfaction and mental health) and performance (grades and number of credit points) over the duration of three quarters of an academic year (8 months) with the actual treatment lasting 2.5 months.

There is a related stream of literature using experiments to study the relationship between social media or computer usage and well-being or performance. Verduyn et al. (2015) conduct a lab experiment where subjects are primed to passively use Facebook for 10 minutes and find that passive use is associated with a decline in subjective well-being. However, it is not straightforward if results from a 10-minute treatment can be generalized to long term effects.

Marotta and Acquisti (2018) conduct an experiment with workers recruited from Amazon mechanical turk and offer productivity enhancing tools to subjects. One of the treatment groups has popular social media sites blocked during work hours. They find that workers in this group completed more tasks and increased their earnings. Carter, Greenberg and Walker (2017) conduct a randomized controlled trial in a US university where classes in the treatment group prohibited the use of computers in the class. They find that average exam scores were higher in the treatment group compared to the control group classes where students were allowed to use their computers. Using causal inference methods on observational data, Belo, Ferreira and Telang (2014) and Beland and Murphy (2016) study the impact of broadband access and banning mobile phones in schools respectively on academic performance and also find evidence suggesting that digital distractions during class reduce academic performance. Taken together, evidence seems to suggest that digital device use in class or at work is harmful for student or worker performance. However, the overall causal impact of social media usage in life (inside and outside class or at work) on performance and well-being still remains an open question. Our study complements this research by analyzing the overall long-term impact of social media on well-being and

performance as the subjects in our treatment group has restricted use of social media throughout their day for a long period of time.

A study closely related to our research is the experiment conducted by Allcott et al. (2020). They conducted a randomized controlled trial of Facebook users where subjects in the treatment group had to deactivate their Facebook account for 1 month. They find that this treatment reduced total online activity including other social media and this reduction persists after the end of the experiment. However, they use self-reported metrics of usage of online activities which are weakly correlated to objective usage metrics according to previous research. They measure 11 different metrics of subjective well-being and find that deactivating Facebook led to increase in subjective well-being for 4 out of the 11 metrics. Other related experiments include Brailovskaia et al. (2020) (two weeks intervention, self-reported Facebook usage measure), Hall et al. (2021) (one to four weeks intervention, self-reported Facebook usage measure), Hinsch and Sheldon (2013) (one week intervention, self-reported Facebook usage measure), Tromholt (2016) (one week intervention, self-reported Facebook usage measure), Mosquera et al. (2020) (one week intervention), Hanley et al. (2019) (one week intervention) and Vanman et al. (2018) (five days intervention). Overall, the magnitude of the effects is small and it is not clear if these effects would have persisted for a treatment of longer duration. For a longer treatment duration, subjects could learn to live in a world without Facebook by discovering alternative substitutes providing similar use cases and their subjective well-being scores could go back to pre-experiment levels.

Methods

Experimental Procedure

We recruited students in the faculty of economics and business of a large European university to take part in an academic study. The study received approval from the Institutional Review

Board of the uUniversity of Groningen. We used a flyer to invite students in lectures and from the pool of participants of the behavioral research lab of the university. The flyer informed students about the subject of the study, the required activities, the reward, and about measures to protect the participants' privacy. Specifically, we let the students know that the study required to install a software (RescueTime) on their computers and mobile devices that keeps track of their digital activities and that allows them to analyze how much time they are spending on various categories of activities. We also stated that the study tracks their academic performance and well-being. Moreover, we informed the students that, in order to qualify for the reward, they need to keep the software running during the time of the study and to take part in four online surveys; one at the beginning of the study and one after each quarter. In addition to getting the software for free, we offered students €20 and a one out of 100 chance to win €1,000 if they take part until the end of the study.

The sign-up link forwarded interested students to a registration form that provided a more detailed privacy statement (about aim and principles of processing personal data, the types of data used, the limited recipients of the data, the storage period, and the students' rights), informed consent, and asked students for their student email address, basic information about their studies (program, year), and the number and type of computers and mobile devices. The registered students were then invited to the study according to the experimental design detailed below.

Experimental Design

The recruitment of students took place in the first quarter of the academic year 2018/19. We scheduled the experiment to run for the remaining three academic quarters. We will refer to these three terms as block 1, 2, and 3 of the study (which are quarter 2, 3, and 4 of the academic year). Each block consists of seven weeks of teaching and two examination weeks. The specific timeline was:

- Block 1: from mid-November to end of January, with holidays from December 24 to January 3.
- Block 2: from February to mid-April.
- Block 3: from mid-April to end of June, with holidays from April 19 to 24.

We used the first block to establish a baseline of the students' digital activities. In block 2, we randomly assigned participants to one of two conditions: a control group without specific instructions and a treatment group that received an incentive to use social media as little as possible. Specifically, we instructed them to use Facebook, Instagram and Snapchat for a maximum of 10 minutes per day. We did not block these services completely because not having access to social media at all might have a negative effect on students[6], e.g., if they use it to exchange important information about their studies. The 10-minute limit enables students to still access relevant information while not allowing them to waste a longer period of time. The software would inform students in the treatment group when they reached the limit and automatically block Facebook, Instagram and Snapchat afterwards. Students could disable this feature if they needed to use these services for longer. We informed students that we gave away another €1,000 among all students who achieved to stay under the 10-minute limit throughout block 2. Block 3 served to assess post-treatment effects.

We focus on Facebook, Instagram, and Snapchat in our experiment because they were the most popular social media services according to our measurement in block 1. As a caveat, the distinction of these services from others such as instant messaging can be debated. We return to this issue in the discussion.

We invited students to four surveys in total. We have sent the first survey in the first week of block 1. This survey asked students to give informed consent and, after referring them to the

[6] This is consistent with the Goldilocks hypothesis according to which moderate digital use may be advantageous compared to no use or overuse (Przybylski and Weinstein 2017).

privacy statement, their agreement to use their academic grades for the purpose of the study. Moreover, we asked them about basic demographic information, their study program, and additional work activities next to their studies. Moreover, we provided measures of subjective well-being (see specific measures below). Upon completion of this first survey, we gave students the installation and registration instructions for the tracking software and asked them to keep this software running henceforth on all their computers and mobile devices[7]. Surveys 2, 3, and 4 followed after each block and repeated the subjective well-being measures in order to track students' well-being over time. We gave students a one-week deadline to fill out each survey.

Survey Measures

As measures of subjective well-being we use the satisfaction with life scale (SWLS; Diener et al., 1985) that consists of five items (In most ways my life is close to my ideal; The conditions of my life are excellent; I am satisfied with my life; So far I have gotten the important things I want in life; If I could live my life over, I would change almost nothing)[8]. These items are measured on a 7-point scale (1 "strongly disagree" to 7 "strongly agree"). SWLS is the most widely used scale to measure subjective well-being and is also used in previous studies studying social media use and well-being (e.g. Kross et al. 2013, Verduyn et al. 2015).

[7] While the software was supported by Windows, OS X, and Android devices, it was not compatible with iOS devices (iPhone or iPad). In order to make sure that students with iOS devices complied to the 10 minute limit in the treatment condition, we informed them that we will ask them at a random time to hand in a screenshot of the Screen Time feature of iOS that reports similar information.

[8] In addition to SWLS, we also collected direct measures of happiness and life satisfaction through standard questions widely used in previous literature. Besides numerous other studies, the happiness question is used in the World values survey (Inglehart et al. 2014) and the life satisfaction question is used by Gallup (Kahneman and Deaton 2010) to calculate its well-being index. These questions are highly correlated with objective measures of well-being such as brain activity, emotional expressions and suicide rates as well as decision utility (Perez-Truglia 2019). We obtain qualitatively similar results using these happiness and life satisfaction scores as we found using SWLS.

For measuring mental well-being, we adopted the shortened Warwick-Edinburgh Mental Well-being Scale (SWEMWBS; Tennant et al., 2007; Stewart-Brown et al. 2009) with seven items (I've been feeling optimistic about the future; I've been feeling useful; I've been feeling relaxed; I've been dealing with problems well; I've been thinking clearly; I've been feeling close to other people; I've been able to make up my own mind about things). These items are assessed on a 5-point scale ranging from "None of the time" to "All of the time". The SWEMWBS is a popular scale to measure mental well-being and is used in previous studies studying technology use and mental well-being (Przybylski and Weinstein 2017).

Overview of Data Sources

Overall, our study makes use of three data sources: digital activities tracked by the software, self-reported measures via surveys, and academic grades from the educational administration. Table 1 shows an overview of these data types.

The software tracks users' activities on each device in 5-minute intervals and records how many seconds a user has actively used a specific program, app, or website in this interval, ranging from 1 to 300 seconds. Specifically, it records the user id, the name of the activity, the system name (Windows, Mac OS, or Android), and a timestamp. Since we are specifically interested in social media activities of the three most used social network services Facebook, Instagram and Snapchat, we used lookup tables to classify activities accordingly. For example, Facebook usage could appear in the activities as "facebook.com", "fb.com", "messenger.com", "Facebook for Android", "Facebook for Windows", etc. We gathered this list of activities in block 1 and used each of these activities to count toward the 10-minute limit for the treatment group in block 2.

The European university at which this study took place uses a grading system that ranges from 0 to 10. Any grade below 6 represents a fail. A grade of 7 is most common and often referred to as "standard", a 6 as "below standard", and an 8 or higher as "above standard".

The grade for a lecture typically consists of a combined grade of the final exam and assignments that have to be completed during the course.

Table 1: Overview of data sources

Type	Measure	Source	Data collection
Digital activities	Usage in number of seconds	Tracked by software	On each participant's device throughout the entire study
Subjective well-being	Rating scales	Self-reported in surveys	At the beginning of the study and after each teaching block
Academic grades	From 0 to 10, with <6 = failed, 6 = below standard, 7 = standard, >8 above standard;	Educational administration	Once at the end of the academic year

Sample

A total of 191 respondents completed the first survey. As is typical for longitudinal studies, some students dropped out over time such that 157 students completed survey 2, 144 survey 3, and 121 the final survey. The survey participation corresponds to the number of participants who reported digital activities using the software (see Table 2).

The following results will be based on the sample that recorded activities for at least 30 days in block 1 and 2 and completed surveys 1, 2, and 3. We will analyze the post-treatment data from block 3 and survey 4 separately. From the 134 students who recorded activities in block 1 and 2, we were able to match 122 from all data sources, i.e., twelve students did not answer (one of) the surveys or did not follow courses in at least one of the blocks.

~~The maximum difference in life satisfaction (on the SWLS scale) that our sample cannot detect is 3 on a scale of 5-35 (average life satisfaction score in our sample is 25 with a standard deviation of 5). A score of 25-29 is considered as a "high score", therefore even if the treatment group's life satisfaction score increased by 3 it is not sufficient to change the classification of the score from "high" to "very high". The maximum difference in average grade that our sample cannot detect is 0.7 on a scale of 1-10 (average grade in our sample is~~

~~about 7 with a standard deviation of 1). Given that students receive grades which are whole numbers, this threshold is still below the value that would increase the treatment group's average grade by a full point.~~

Despite the dropouts, most importantly, there are no significant differences between the treatment and the control group, in terms of gender (Chi-squared test, p = 0.471), age (t-test, p = 0.961), mobile device operating system (Chi-squared test, p = 1.000), number of years studying at the university (t-test, p = 0.334) or whether students are working next to their studies in block 1 (Chi-squared test, p = 0.974) or block 2 (p = 0.594) (see Table 3 for details). There are also no significant differences between those who started the study and those who dropped out in terms of gender (Chi-squared test, p = 0.701), age (t-test, p = 0.113), mobile operation system (Chi-squared test, p = 0.975), of work status in block 1 (Chi-squared test, p = 0.109) or block 2 (p = 0.169). However, there is a significant difference between these samples regarding the study year (t-test, p = 0.027) such that those who dropped out are more likely to be Bachelor degree students than Master's students. One potential explanation is that Bachelor degree students are more likely to quit their studies and not have any courses or grades registered subsequently.

For the subsequent analysis, we note that at 80% power and alpha = 5%, the minimum detectable difference with our sample size is 2.5 for SWLS (assuming a standard deviation of 5.0), 1.5 for SWEMWBS (standard deviation = 3.0), and 0.5 for academic grades (standard deviation of 1.0).

Table 2: Number of participants

Part	Number
Completed survey 1	191
Used software in block 1 (calibration)	149

Completed survey 2	157
Used software in block 2 (treatment)	134
Completed survey 3	144
Used software in block 3 (post-treatment)	125
Completed survey 4	121
Took courses in block 1, 2, and 3	158

Table 3: Descriptive statistics of the sample

	Treatment	Control
Number of students	60	62
Gender: Female (vs. male)	0.467	0.548
Age (SD)	22.1 (3.3)	22.1 (3.1)
Mobile device operating system: Android (vs. iOS)	0.500	0.500
Studying in first to third year (vs. more than three years)	0.667	0.629
Working next to studying (block 1)	0.400	0.419
Working next to studying (block 2)	0.500	0.435

Results

Digital Activities

Social media usage

On average, students tracked 223.7 minutes of digital activities per day across the entire study (SD = 115.1 minutes). Students who use an Android mobile device recorded significantly more activities (265.6 minutes; t-test, $p < 0.001$), compared to students with an iOS device (182.5 minutes) as iOS was not supported by the software. While our activity estimates are more accurate for Android users we expect the treatment condition to be equally effective for both of these segments because we informed participants to also inspect their iOS tracked activities (see above).

Figure 1 shows the total number of minutes tracked by day, averaged for students with Android mobile devices in the treatment (black dots) and control groups (white dots). We report the activities for users with Android devices because the tracking is more accurate as it captures activities on desktop/laptop computers and their mobile devices (figures corresponding to the overall sample are in the Supporting Information, Figure S1). The solid vertical lines separate blocks 1, 2, and 3 and the dashed vertical lines indicate the start of the examination period. Overall, digital activities remain on a high level each day but are reduced during the winter holiday season and during the examination periods.

As a manipulation check, the bottom part of Figure 1 shows activities for social networking (Facebook, Instagram, and Snapchat combined) for the Android sample. The mean daily usage in minutes is 21.1 minutes (27.9 minutes) for users in the treatment (control) group in block 1, which is not significantly different (t-test, $p = 0.310$). The incentive to reduce social media activities was effective as students in the treatment condition significantly reduced their social media usage in block 2 compared to the control group (t-test, $p = 0.009$). The

horizontal line represents the 10-minute limit imposed on the treatment group. The average usage per day is close to the limit in the treatment group with 8.1 minutes. Within the control group, the average daily usage of 24.2 minutes in block 2 is on the same level as in the first block (paired-sample t-test, p = 0.245).

Figure 1: All digital activities and social media usage over time (users with Android devices)

Remarkably, although students in the treatment group significantly reduced their social media activities, their overall digital activities overall are not affected but, in fact, exceed those of the control group in block 2 (t-test, p = 0.026). This result indicates that students substituted or even overcompensated their social media usage with other activities.

Substitution. Figures 2.1 and 2.2 show the time series of activities of users with an Android device for the most used categories of services (we exclude the categories of general utilities, which holds mostly operating system activities, and uncategorized services for which there are no significant differences between the groups; activities for all users are in the Supporting Information, Figures S2.1 and S2.2). We find significant substitution for social networking with instant messaging (p = 0.008). Accordingly, more students in the treatment condition used instant messaging in block 2 when social media was restricted compared to block 1 and to the control group (difference-in-differences). The activities increased from an average daily use of 25.1 minutes in block 1 to 28.8 minutes in block 2 in the treatment group, while the usage decreased from 21.8 minutes to 15.2 minutes in the control group. Most activities (92.9%) in this category are related to WhatsApp.

We also find a significant increase in usage of music for Android users (t-test, p = 0.027) in the treatment group in block 2. However, average daily activities in this category are rather

low (below five minutes) and the difference is mostly driven by two outliers who listen to music for more than 30 minutes each day on average. Other activities show plausible patterns, e.g., the reference and learning category (activities include the university intranet, PDF reader, Wikipedia, Mendeley, Google scholar, EBSCO, etc.) shows peaks before the exam period. However, these and other activities are not affected by reduced social media usage (an overview of significance tests comparing treatment and control groups in block 2 vs. block 1 is given in the Supporting Information, Table S1).

Figure 2.1: Tracked digital activities over time (users with Android devices)

Figure 2.2: Tracked digital activities over time (users with Android devices)

Subjective Well-being

For the subjective well-being measures, the SWLS and SWEMWBS items show high reliability (Cronbach's α being 0.84 and 0.76 respectively). For the analysis we calculate scores as the sum of their items (with SWEMWBS being transformed according to a defined conversion table). The students score averages (SD) in SWLS of 25.0 (5.5), 25.0, (5.4), 25.1 (5.3) in the three surveys at the beginning of the study and after block 1 and 2. That means they are between an "average" and "high" score of satisfaction. Treatment and control group are not significantly different at the beginning of block 1 (t-test, p = 0.182; survey 1), at the end of block 1 (t-test, p = 0.212; survey 2), or, most importantly, at the end of block 2 after the exposure to the treatment (t-test, p = 0.167; survey 3). The same implications hold for the SWEMWBS scores that shows average scores (SD) of 22.8 (3.0), 22.5 (2.7), and 22.4 (3.3) in the three surveys (see Table S2 in the Supporting Information for details).

Figure 3 plots the differences between survey 3 and survey 2 (before and after the social media restriction) in terms of SWLS and SWEMBS. The distributions are centered on zero, illustrating the non-significant difference between the treatment and control group.

Figure 3: Differences in subjective well-being measures

Table 4 shows correlations with the subjective well-being measures and the digital activities in block 1 (i.e., activities that are not affected by the treatment condition) for users with an Android device. We detail the correlations of the subjective well-being measures at the beginning of the first block (in survey 1) and at the end of the block (in survey 2) to study potential reverse causality, e.g., increased well-being leads so more/less social media activities or vice versa.

Satisfaction with life and mental well-being are positively correlated and are also significant predictors over time, i.e., subjective well-being in survey 1 is positively correlated with the same measure in survey 2. Regarding digital activities, we see, on average, negative correlations between subjective well-being and all digital activities, albeit not being significant. Similarly, we do not find significant correlations with social media use. (We will address causality in the section below.) To address potential non-linear effects we also report correlations with categories of social media usage. Specifically, we used dummy variables relating to low usage with an average of less than 2 minutes per day (36.1% of users), medium usage of 2 to 20 minutes (39.5%), and high usage of 20 minutes or more (24.4%). A non-linear relationship is likely as low usage generally shows the most negative subjective well-being scores, while medium usage and not high usage scores the highest well-being. However, we cannot rule out reverse causality regarding these findings as the only significant

relationships are between satisfaction with life measured in survey 1 and the social media activities measured after the survey has taken place.

Activities related to communication, i.e., instant messaging and email, show significant positive correlations in the second surveys (for instant messaging regarding mental well-being and for email in terms of satisfaction with life). A consistent significant negative correlation can be observed for activities in the video category and satisfaction with life (both surveys) and mental well-being (survey 1). This suggests that less satisfied students and those with lower mental well-being at the beginning of the block increasingly watch videos in the subsequent block.

Table 4: Correlations of subjective well-being measures and digital activities in block 1 (Android users)

	SWLS Survey 1 (start of block 1)	SWLS Survey 2 (end of block 1)	SWEMWBS Survey 1 (start of block 1)	SWEMWBS Survey 2 (end of block 1)
SWLS Survey 2 (end of block 1)	**0.80**			
SWEMWBS Survey 1 (beginning of block 1)	**0.76**	**0.63**		
SWEMWBS Survey 2 (end of block 1)	**0.63**	**0.72**	**0.77**	
All digital activities	-0.06	-0.11	-0.15	-0.05
Social media usage	-0.03	-0.02	-0.13	-0.02
Social media usage low	**-0.31**	-0.23	-0.08	-0.13
Social media usage medium	**0.26**	0.19	0.14	0.15
Social media usage high	-0.02	-0.01	-0.08	-0.05
General Reference & Learning	-0.13	-0.14	-0.20	-0.06
Instant Message	0.18	0.24	0.14	**0.27**
Browsers	-0.09	-0.13	-0.01	-0.11
Video	**-0.28**	**-0.27**	**-0.28**	-0.21
Writing	0.24	**0.27**	0.10	0.09
Search	-0.19	-0.19	-0.22	-0.10
Email	0.21	**0.28**	0.11	0.10
General News & Opinion	0.19	0.04	0.14	0.13

Games	0.06	-0.17	0.04	-0.04
Presentation	0.14	0.10	-0.01	0.03
General Shopping	-0.05	0.02	-0.12	0.02
Music	0.12	0.10	0.12	0.24

(Correlations in bold font are significant on a 5% level)

Academic Performance

The students participating in our study scored an average (SD) of 7.105 (1.078) in block 1 and 7.122 (0.954) in block 2. Most grades can be classified as "standard". The average (SD) sum of credit points per block is 13.571 (5.754) in block 1 and 12.353 (5.118) in block 2. Differences between the treatment and control group are not significant for grades (t-test, $p = 0.113$) but for the number of credit points such that the treatment group attempted to score significantly more credits (t-test, $p = 0.035$). This is visualized in Figure 4 as the difference in grades and credits in block 1 and 2. Note that the number of ECTS represents the courses that the student *attempted* to pass but they are also stored if the student failed the exam. A comparison of the number of successfully passed courses shows no significant differences between the groups (t-test, $p = 0.383$).

Figure 4: Differences in academic performance

Table 5 shows the correlations of academic performance in block 1 with the subjective well-being measures and digital activities. Accordingly, grades in block 1 are positively related to grades in block 2. The grades are not significantly correlated with the number of credit points, possibly due to a trade-off regarding a good grade and completing more courses. Credits are also not positively related over time, which is reasonable as more credits in one term means that the students have to obtain fewer credits in subsequent terms. The average

grade is positively and significantly correlated with satisfaction with life measures. This holds for SWLS measures at the beginning[9] and end of the block.

Regarding correlations with digital activities we can observe significant positive effects on the average grade for writing and presentation activities, which are required to complete assignments (that are part of the grade for the majority of courses). We also see a positive effect of instant messaging on the grade, however, a negative effect on the number of credit points. Social media usage is not significantly correlated with the academic performance in block 1. Only when categorizing students based on their social media usage we see significant effects such that medium usage is negatively correlated with the number of credits.

To what extent these findings can be interpreted as causal evidence will be addressed in the following section by analyzing the complete randomized control trial across the two blocks using difference-in-differences analyses with regression models.

Table 5: Correlations of academic performance with measures of subjective well-being and digital activities in block 1 (Android users)

	Average grade in block 1	Sum of credit points in block 1
Sum of credit points in block 1	-0.16	
Average grade in block 2	**0.46**	-0.19
Sum of credit points in block 2	0.06	0.01
SWLS Survey 1 (beginning of block 1)	**0.27**	-0.10
SWLS Survey 2 (end of block 1)	**0.31**	0.02
SWEMWBS Survey 1 (beginning of block 1)	0.16	-0.07
SWEMWBS Survey 2 (end of block 1)	0.22	0.01
All digital activities	0.16	-0.16
Social media usage	0.02	-0.03

[9] We have also obtained grades for the first quarter (i.e., block 0). These grades are also significantly correlated with SWLS measures in Survey 1 such that a reasonable explanation would be that academic grades positively affected subjective well-being measured in the subsequent survey.

Social media usage low	-0.08	0.21
Social media usage medium	0.06	**-0.26**
Social media usage high	0.00	0.09
General Reference & Learning	0.13	-0.21
Instant Message	**0.28**	**-0.28**
Browsers	-0.03	-0.18
Video	-0.04	0.15
Writing	**0.25**	0.05
Search	-0.13	-0.21
Email	0.12	-0.21
General News & Opinion	-0.07	-0.10
Games	-0.06	-0.08
Presentation	**0.28**	0.17
General Shopping	-0.02	-0.01
Music	-0.08	-0.13

(Correlations in bold font are significant on a 5% level)

Regressions

Tables 6 and ~~6~~ 7 show~~s~~ the results of the~~a~~ regression models that use subjective well-being (Table 6) and academic performance measures (Table 7) as the dependent variables. ~~of the average grade and number of credit points.~~ We use data from the two teaching blocks with "block 2" being a dummy variable indicating the block in which the treatment took place. Similarly, the "treatment group" refers to a dummy variable that identifies students that were exposed to the treatment. We use gender, age, number of years at the university, and whether the student is working next to the studies (dummy variable) as control variables.

Regarding subjective well-being measures SWLS and SWEMWBS, we do not see any significant effects due to using Facebook, Instagram, and Snapchat less. The ~~s~~(Treatment group * Block2 interaction) that indicates if the treatment group differed from the control group in block 2 remains insignificant, irrespectively of the well-being measure (SWLS or SWEMWBS) or sample used (full sample or Android subset). Only few predictors are significant (gender in the full sample, $p = 0.013$ or age in the Android subset, $p = 0.026$; both

for the SWEMWBS) but these are not directly related to the (see Supporting Information Table S3).experimental setting. Overall, the amount of variance explained remains rather low and ranges between 3.2% (SWLS measure, full sample) and 14.2% (SWEMWBS, Android sample).

There is also no evidence that the treatment group achieves higher grades in block 2 (Treatment group * Block2 interaction), in which social media usage was restricted, than the control group ($p = 0.239$). This also holds for other subsets of the sample such as students with an Android device. Overall, the amount of variance explained in the grades remains very low with 3.4%. Only the number of years that the student has spent at the university is a significant positive predictor, i.e., Master's students achieve higher grades than Bachelor's. In terms of number of credit points, i.e., number of courses attended, we do see a significant effect of restricting social media. While students overall attended courses with fewer credits in block 2 ($p = 0.004$) this is not the case for students in the treatment group such that they targeted significantly more credits ($p = 0.023$). With this dependent variable, the number of years at the university has a significant negative effect ($p = 0.002$) and, overall, 11.7% of variance in the number of credit points can be explained. The subset of Android users replicates these results, albeit generally with lower levels of significance due to the smaller sample size.

However, as noted above, the number of credit points does not necessarily show that students successfully passed more courses as also failed courses are included. Using the number of courses passed as the dependent variable shows that the students in the treatment group in fact do not differ from the control group (a Poisson model replicates these results). Thus, it appears that the treatment group attempted to pass more courses or courses with more credits compared to the control group but did not necessarily succeed.

~~Regarding subjective well-being measures SWLS and SWEMWBS, we do not see any significant effects due to using Facebook, Instagram, and Snapchat less (see Supporting Information Table S3).~~

Table 6: Regression of satisfaction with life and mental well-being measures (standardized coefficients in parentheses)

	Satisfaction with life (SWLS)				Mental well-being (SWEMWBS)			
	All users		Android		All users		Android	
	Estimate	p-value	Estimate	p-value	Estimate	p-value	Estimate	p-value
(Intercept)	**29.028**	**<0.001**	**36.967**	**<0.001**	**24.409**	**<0.001**	**30.497**	**<0.001**
Treatment group	-1.205 (-0.113)	0.221	-1.350 (-0.117)	0.380	-0.233 (-0.038)	0.676	-0.063 (-0.009)	0.942
Block 2	0.207 (0.019)	0.831	-0.552 (-0.048)	0.720	0.025 (0.004)	0.963	-0.342 (-0.050)	0.691
(Treatment group*Block2)	-0.253 (-0.020)	0.855	1.285 (0.097)	0.552	-0.126 (-0.018)	0.872	0.505 (0.065)	0.676
Gender (female)	-1.021 (-0.096)	0.143	-0.818 (-0.070)	0.455	**-0.991 (-0.162)**	**0.013**	-0.768 (-0.112)	0.210
Age in years	-0.161 (-0.096)	0.221	-0.494 (-0.217)	0.068	-0.057 (-0.059)	0.446	**-0.338 (-0.252)**	**0.026**
Years at the university	0.166 (0.048)	0.542	0.107 (0.028)	0.798	-0.139 (-0.069)	0.370	-0.089 (-0.040)	0.705
Working next to studies	0.280 (0.026)	0.692	-0.036 (-0.003)	0.975	0.724 (0.117)	0.072	1.020 (0.150)	0.117
R-squared	0.032		0.051		0.055		0.142	

Table 76: Regression of academic performance (standardized coefficients in parentheses)

	Regression of average grade				Regression of number of credit points				Regression of number of courses passed			
	All users		Android users		All users		Android users		All users		Android users	
	Estimate	p-value	Estimate	p-value	Estimate	p-value	Estimate	p-value	Estimate	p-value	Estimate	p-value
(Intercept)	**6.950**	**<0.001**	**6.578**	**<0.001**	**19.406**	**<0.001**	**13.393**	**0.009**	**3.904**	**<0.001**	**2.616**	**0.007**
Treatment group	0.237 (0.117)	0.205	0.536 (0.244)	0.067	-1.740 (-0.159)	0.071	-1.159 (-0.108)	0.388	-0.064 (-0.032)	0.721	0.124 (0.064)	0.625
Block 2	0.173 (0.086)	0.350	0.321 (0.146)	0.272	**-2.750 (-0.252)**	**0.004**	**-2.931 (-0.273)**	**0.031**	-0.347 (-0.172)	0.051	-0.345 (-0.177)	0.178
(Treatment group*Block2)	-0.310 (-0.132)	0.239	-0.592 (-0.235)	0.149	**3.090 (0.245)**	**0.023**	3.431 (0.278)	0.070	0.220 (0.094)	0.382	0.211 (0.094)	0.554
Gender (female)	0.142 (0.070)	0.281	0.137 (0.062)	0.507	0.706 (0.065)	0.300	0.388 (0.036)	0.684	0.201 (0.099)	0.113	0.090 (0.046)	0.618
Age in years	-0.016 (-0.048)	0.534	0.002 (0.005)	0.969	-0.130 (-0.076)	0.311	0.164 (0.078)	0.483	**-0.055 (-0.172)**	**0.022**	0.003 (0.007)	0.949
Years at the university	**0.114** (0.172)	**0.028**	0.083 (0.116)	0.294	**-0.854 (-0.239)**	**0.002**	**-1.232 (-0.352)**	**0.001**	**-0.109 (-0.165)**	**0.028**	-0.131 (-0.206)	0.060
Working next to studies	-0.043 (-0.021)	0.750	-0.202 (-0.092)	0.358	-0.009 (-0.001)	0.990	1.439 (0.134)	0.156	-0.082 (-0.040)	0.523	0.134 (0.069)	0.484
R-squared	0.034		0.054		0.117		0.159		0.113		0.083	

These null results of the effects of restricting social media usage on academic performance and subjective well-being raises the question of whether our study is underpowered to detect economically significant effects. At 80% power the maximumminimum detectable difference in life satisfaction (on the SWLS scale) thatwith our sample cannotsize detect is 2.53 on a scale of 5-35 (average life satisfaction score in our sample is 25 with a standard deviation of 5). The SWLS categories comprise 5 scale points, for example, a score of 25-29 is considered as a "high" score. Therefore, even if the treatment group's life satisfaction score increased by 1.53 it is generally not sufficient to change the classification from one category to another. The maximumminimum detectable difference in average grade that our sample cannot detect is 0.57 on a scale of 1-10 (average grade in our sample is about 7 with a standard deviation of 1). Given that students receive grades which are whole numbers, this threshold is still below the value that would increase the treatment group's average grade by a full point. The maximum difference in life satisfaction (on the SWLS scale) that our sample cannot detect is 3 on a scale of 5-35 (average life satisfaction score in our sample is 25 with a standard deviation of 5). A score of 25-29 is considered as a "high score", therefore even if the treatment group's life satisfaction score increased by 3 it is not sufficient to change the classification of the score from "high" to "very high". The maximum difference in average grade that our sample cannot detect is 0.7 on a scale of 1-10 (average grade in our sample is about 7 with a standard deviation of 1). Given that students receive grades which are whole numbers, this threshold is still below the value that would increase the treatment group's average grade by a full point. At least, our results indicate that changes in grades and subjective well-being are not substantial so that they could be detected with our sample size. To further address potential concerns about statistical power we applied a hierarchical Bayes ANOVA (BANOVA) model that includes between and within subject effects and accommodates unobserved heterogeneity by including a normal distribution of the

parameters across individuals (Wedel and Dong 2019). All models converge and generally replicate the results above (details are available from the authors upon request).

Post-treatment Effects

The formal analysis of the post-treatment effects is based on a sample of 106 students who provided activity data throughout all three blocks and in all four surveys. While the treatment condition significantly reduced their social media usage in block 2 compared to the control group, this effect was not permanent. After we suspended the limit in block 3 the social media activities of users in the treatment group increased again, showing no significant differences to the control group any longer (t-test, $p = 0.668$). We further do not see any significant differences between the treatment and control group in block 3 in terms of grades (t-test, $p = 0.152$), number of credit points (t-test, $p = 0.923$), satisfaction with life (t-test, $p = 0.499$), or mental well-being (t-test, $p = 0.966$), i.e., there is no lagged effect of reduced social media usage.

Discussion

In this paper, we analyzed the effects of restricting social media usage. We did not find significant causal effects of social media usage on well-being or academic performance, other than students attempting (but not succeeding) to pass more courses or courses with more credits. However, we found robust evidence of substitution effects that can potentially explain the null finding. Specifically, we showed that social media and instant messaging apps can be substitutes. The European Commission approved Facebook's acquisition of WhatsApp in 2014 based on Facebook's claim that it operates in a different market and does not compete directly with WhatsApp[10]. Our results indicate that they are in fact direct competitors. After acquiring WhatsApp, Facebook started automatically matching its users'

[10] Source: European Commission (http://europa.eu/rapid/press-release_IP-14-1088_en.htm, accessed on June 1, 2019)

profiles with their WhatsApp accounts[11] and has started integrating WhatsApp, Instagram and Facebook user accounts[12]. Antitrust authorities should consider the market power of this combined entity if the world's biggest social media platforms are integrated with the world's biggest instant messaging platform. This finding also raises the question how to properly define social networks, for future academic studies or antitrust cases.

While we found null results estimating the causal impact of social media usage on well-being and academic performance, and not all null results matter, we believe that null results are interesting and important in this context. The media has hyped correlational studies showing a negative association between social media usage and well-being and it is important to balance this narrative through causal evidence. A limitation of our study is the lack of a larger sample size to detect smaller effects. While these small effects might not be economically significant, more research is needed using massive samples.[13] However, it is challenging to recruit a large number of subjects from a representative sample for a long-term study. Direct collaborations with social media platforms or internet service providers (which control internet traffic) could be a way of obtaining data from larger samples. These would also facilitate more targeted interventions such as restricting only content consumption or social interactions on social media (Liu et al. 2019).

It is interesting to notice that while social media generates large amount of consumer surplus (Brynjolfsson, Collis and Eggers 2019), it doesn't seem to affect the subjective well-being of users. Future research can explore this wedge between consumer surplus and subjective well-

[11] The European Commission fined Facebook €110 million in 2017 for this practice because Facebook had provided misleading information about the feasibility of automatically matching profiles during its acquisition of WhatsApp. However while announcing this fine, the Commission still maintained its belief that Facebook and WhatsApp do not directly compete with each other (http://europa.eu/rapid/press-release_IP-17-1369_en.htm, accessed on June 1, 2019).

[12] Source: The New York Times (https://www.nytimes.com/2019/01/25/technology/facebook-instagram-whatsapp-messenger.html, accessed on June 1, 2019; https://www.nytimes.com/2020/09/30/technology/instagram-facebook-messenger-integration.html, accessed on October 19, 2020).

[13] Future research can also look at differences between Android and iOS users in more detail with larger samples since we lack power to analyze these differences in our current study.

being and see whether they are correlated for some products and uncorrelated for others. Future research should also explore the addictiveness of social media in more detail (Allcott et al. 2021). Our findings in block 3 show that the students in the treatment condition go back to their old habits and do not adopt a lower social media usage that they experienced in block 2. On the other hand, showing students how much time they are spending on social networks via the software seems to have an overall negative trend on its usage (comparing usage in block 1 and block 3). Curing social media addiction (if it is indeed addiction) might therefore be a longer process. Future research can look at mechanisms for the emergence of social media addiction, for e.g. through targeted advertising or news feed algorithms and features of social media apps (e.g. video sharing) which are correlated with addiction.

Moreover, due to our student sample implications for the general population are limited. It could be that students use social media mostly for communication purposes and therefore show significant substitution effects with instant messaging. We might see different effects for users who visit social media for content consumption, e.g., watching videos, instead of social interaction (Liu et. al (2019)). In this regard, newer social media services such as TikTok might have a different effect. However, these and other comparable social media services were not relevant at the time we conducted the study (as we measured in block 1). More research is needed looking at emerging social media platforms such as TikTok. Moreover, one could define social media more broadly to include instant messaging services and implement a stricter restriction involving all types of communication and social networking apps. However, conducting such a study for any length of time is a challenging endeavor.

Additionally, while we study the impact of social media on students and academic performance, future research can look at workplace settings and study the impact of social media and its substitutes on worker productivity and well-being. We believe that rigorous

causal evidence through randomized controlled trials and objectively measured time spent is the way forward in addressing questions regarding the impact of technology on well-being. The widespread adoption of most major technologies in the past such as radio, television, video games and computers was followed with unfounded fears about their impact on well-being. This story repeats again with social media. We find that social media usage does not cause lower well-being or poor academic performance. Rather, we demonstrate that students find other means of social networking using instant messaging when exogenously restricting their social media usage. To conclude: You can take social networking away from the students, but you cannot take students away from their social network.

References

Allcott, H., Braghieri, L., Eichmeyer, S., & Gentzkow, M. (2020). The welfare effects of social media. *American Economic Review*, *110*(3), 629-76.

Allcott, H., Gentzkow, M., & Song, L. (2021). Digital addiction (No. w28936). National Bureau of Economic Research.

Appel, M., Marker, C., & Gnambs, T. (2020). Are social media ruining our lives? A review of meta-analytic evidence. Review of General Psychology, 24(1), 60-74.

Beland, L. P., & Murphy, R. (2016). Ill communication: technology, distraction & student performance. *Labour Economics*, 41, 61-76.

Belo, R., Ferreira, P., & Telang, R. (2013). Broadband in school: Impact on student performance. *Management Science*, 60(2), 265-282.

Brailovskaia, J., Ströse, F., Schillack, H., & Margraf, J. (2020). Less Facebook use–More well-being and a healthier lifestyle? An experimental intervention study. *Computers in Human Behavior*, *108*, 106332.

Formatted: English (United States)

Formatted: German (Germany)

Formatted: English (United States)

Formatted: English (United States)

Brynjolfsson, E., Collis, A., & Eggers, F. (2019). Using massive online choice experiments to measure changes in well-being. *Proceedings of the National Academy of Sciences*, 116(15), 7250-7255.

Burke, M., & Kraut, R. E. (2016). The relationship between Facebook use and well-being depends on communication type and tie strength. *Journal of computer-mediated communication*, 21(4), 265-281.

Burke, M., Marlow, C., & Lento, T. (2010, April). Social network activity and social well-being. In *Proceedings of the SIGCHI conference on human factors in computing systems* (pp. 1909-1912). ACM.

Carter, S. P., Greenberg, K., & Walker, M. S. (2017). The impact of computer usage on academic performance: Evidence from a randomized trial at the United States Military Academy. *Economics of Education Review*, 56, 118-132.

Cheng, J., Burke, M., & Davis, E. G. (2019, April). Understanding Perceptions of Problematic Facebook Use: When People Experience Negative Life Impact and a Lack of Control. In *Proceedings of the 2019 CHI Conference on Human Factors in Computing Systems* (p. 199). ACM.

Diener, E., Emmons, R. A., Larsen, R. J., & Griffin, S. (1985). The Satisfaction with Life Scale. *Journal of Personality Assessment*, 49, 71-75.

Dienlin, T., & Johannes, N. (2020). The impact of digital technology use on adolescent well-being. *Dialogues in clinical neuroscience*, *22*(2), 135.

Ellis, D. A. (2019). Are smartphones really that bad? Improving the psychological measurement of technology-related behaviors. *Computers in Human Behavior*.

Ellis, D. A., Davidson, B. I., Shaw, H., & Geyer, K. (2019). Do smartphone usage scales predict behavior?. *International Journal of Human-Computer Studies*.

Haidt, J. (2019). Social Media Use and Mental Health: A Review. Available at: https://docs.google.com/document/d/1w-HOfseF2wF9YIpXwUUtP65-olnkPyWcgF5BiAtBEy0/edit (accessed on May 11, 2019).

Hall, J. A., Xing, C., Ross, E. M., & Johnson, R. M. (2021). Experimentally manipulating social media abstinence: results of a four-week diary study. *Media Psychology*, *24*(2), 259-275.

Hanley, S. M., Watt, S. E., & Coventry, W. (2019). Taking a break: The effect of taking a vacation from Facebook and Instagram on subjective well-being. *Plos one*, *14*(6), e0217743.

Hinsch, C., & Sheldon, K. M. (2013). The impact of frequent social Internet consumption: Increased procrastination and lower life satisfaction. *Journal of Consumer Behaviour*, *12*(6), 496-505.

Hobbs, W. R., Burke, M., Christakis, N. A., & Fowler, J. H. (2016). Online social integration is associated with reduced mortality risk. *Proceedings of the National Academy of Sciences*, 113(46), 12980-12984.

Huang, C. (2017). Time spent on social network sites and psychological well-being: A meta-analysis. *Cyberpsychology, Behavior, and Social Networking*, *20*(6), 346-354.

Inglehart, R., C. Haerpfer, A. Moreno, C. Welzel, K. Kizilova, J. Diez-Medrano, M. Lagos, P. Norris, E. Ponarin & B. Puranen et al. (eds.). 2014. World Values Survey: All Rounds - Country-Pooled Datafile 1981-2014. Madrid: JD Systems Institute.

Junco, R. (2013). Comparing actual and self-reported measures of Facebook use. *Computers in Human Behavior*, 29(3), 626-631.

Johannes, N., Meier, A., Reinecke, L., Ehlert, S., Setiawan, D. N., Walasek, N., ... & Veling, H. (2020). The relationship between online vigilance and affective well-being in

everyday life: Combining smartphone logging with experience sampling. *Media Psychology*, 1-25.

Kahneman, D., & Deaton, A. (2010). High income improves evaluation of life but not emotional well-being. *Proceedings of the national academy of sciences*, 107(38), 16489-16493.

Kim, C., & Shen, C. (2020). Connecting activities on Social Network Sites and life satisfaction: A comparison of older and younger users. *Computers in Human Behavior*, *105*, 106222.

Kraut, R., & Burke, M. (2015). Internet use and psychological well-being: Effects of activity and audience. *Communications of the ACM*, 58(12), 94-100.

Kross, E., Verduyn, P., Demiralp, E., Park, J., Lee, D. S., Lin, N., ... & Ybarra, O. (2013). Facebook use predicts declines in subjective well-being in young adults. *PloS one*, 8(8), e69841.

Liu, D., Baumeister, R. F., Yang, C. C., & Hu, B. (2019). Digital communication media use and psychological well-being: A meta-analysis. *Journal of Computer-Mediated Communication*, *24*(5), 259-273.

Marotta, V., & Acquisti, A. (2018). Interrupting Interruptions: A Digital Experiment on Social Media and Performance. Available at SSRN 3283951.

Masur, P. K. (2021). Digital Communication Effects on Loneliness and Life Satisfaction. In *Oxford Research Encyclopedia of Communication*.

Meier, A., & Reinecke, L. (2020). Computer-mediated communication, social media, and mental health: A conceptual and empirical meta-review. Communication Research, 0093650220958224.

Mosquera, R., Odunowo, M., McNamara, T., Guo, X., & Petrie, R. (2020). The economic effects of Facebook. *Experimental Economics*, *23*(2), 575-602.

Orben, A. (2020). Teenagers, screens and social media: a narrative review of reviews and key studies. *Social psychiatry and psychiatric epidemiology*, *55*(4), 407-414.

Orben, A., Dienlin, T., & Przybylski, A. K. (2019). Social media's enduring effect on adolescent life satisfaction. *Proceedings of the National Academy of Sciences*, 116(21), 10226-10228.

Orben, A., & Przybylski, A. K. (2019). The association between adolescent well-being and digital technology use. *Nature Human Behaviour*, 3(2), 173.

Perez-Truglia, R. (2019). The effects of income transparency on well-being: Evidence from a natural experiment (No. w25622). National Bureau of Economic Research.

Przybylski, A. K., & Weinstein, N. (2017). A large-scale test of the Goldilocks Hypothesis: Quantifying the relations between digital-screen use and the mental well-being of adolescents. *Psychological Science*, 28(2), 204-215.

Scharkow, M. (2016). The accuracy of self-reported Internet use—A validation study using client log data. *Communication Methods and Measures*, 10(1), 13-27.

Schemer, C., Masur, P. K., Geiß, S., Müller, P., & Schäfer, S. (2021). The impact of internet and social media use on well-being: A longitudinal analysis of adolescents across nine years. *Journal of Computer-Mediated Communication*, *26*(1), 1-21.

Sewall, C. J., Goldstein, T. R., Wright, A. G., & Rosen, D. (2022). Does Objectively Measured Social-Media or Smartphone Use Predict Depression, Anxiety, or Social Isolation Among Young Adults?. *Clinical Psychological Science*, 21677026221078309.

Shakya, H. B., & Christakis, N. A. (2017). Association of Facebook use with compromised well-being: A longitudinal study. *American journal of epidemiology*, 185(3), 203-211.

Stewart-Brown, S., Tennant, A., Tennant, R., Platt, S., Parkinson, J., & Weich, S. (2009). Internal construct validity of the Warwick-Edinburgh mental well-being scale

Formatted: Font: Italic

(WEMWBS): a Rasch analysis using data from the Scottish health education population survey. *Health and quality of life outcomes*, 7(1), 15.

Tennant, R., Hiller, L., Fishwick, R., Platt, S., Joseph, S., Weich, S., ... & Stewart-Brown, S. (2007). The Warwick-Edinburgh mental well-being scale (WEMWBS): development and UK validation. *Health and Quality of life Outcomes*, 5(1), 63.

Tromholt, M. (2016). The Facebook experiment: Quitting Facebook leads to higher levels of well-being. *Cyberpsychology, behavior, and social networking*, *19*(11), 661-666.

Vanman, E. J., Baker, R., & Tobin, S. J. (2018). The burden of online friends: The effects of giving up Facebook on stress and well-being. *The Journal of social psychology*, *158*(4), 496-508.

Verduyn, P., Lee, D. S., Park, J., Shablack, H., Orvell, A., Bayer, J., ... & Kross, E. (2015). Passive Facebook usage undermines affective well-being: Experimental and longitudinal evidence. *Journal of Experimental Psychology: General*, 144(2), 480.

Wedel, M., Dong, C. (2019). BANOVA: Bayesian Analysis of Experiments in Consumer Psychology. *Journal of Consumer Psychology*, 1– 21.

Figures

Figure 1: All digital activities and social media usage over time (users with Android devices)

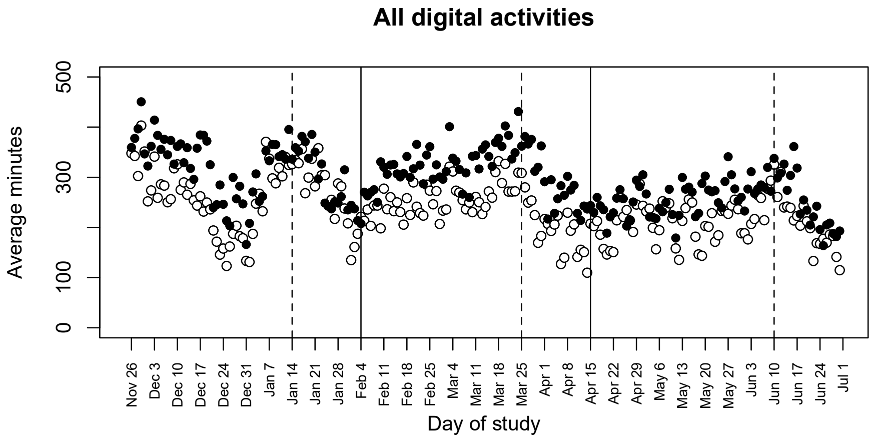

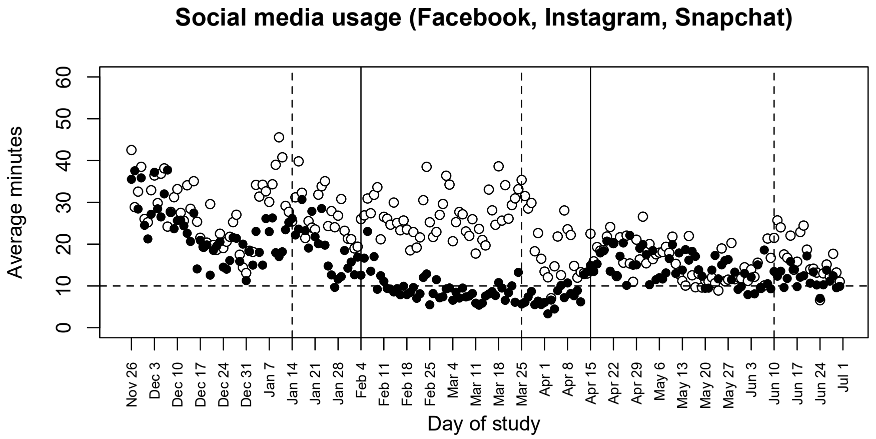

black = treatment, white = control group
solid vertical lines: start of a new teaching block
dashed vertical lines: start of the exam period

Figure 2.1: Tracked digital activities over time (users with Android devices)

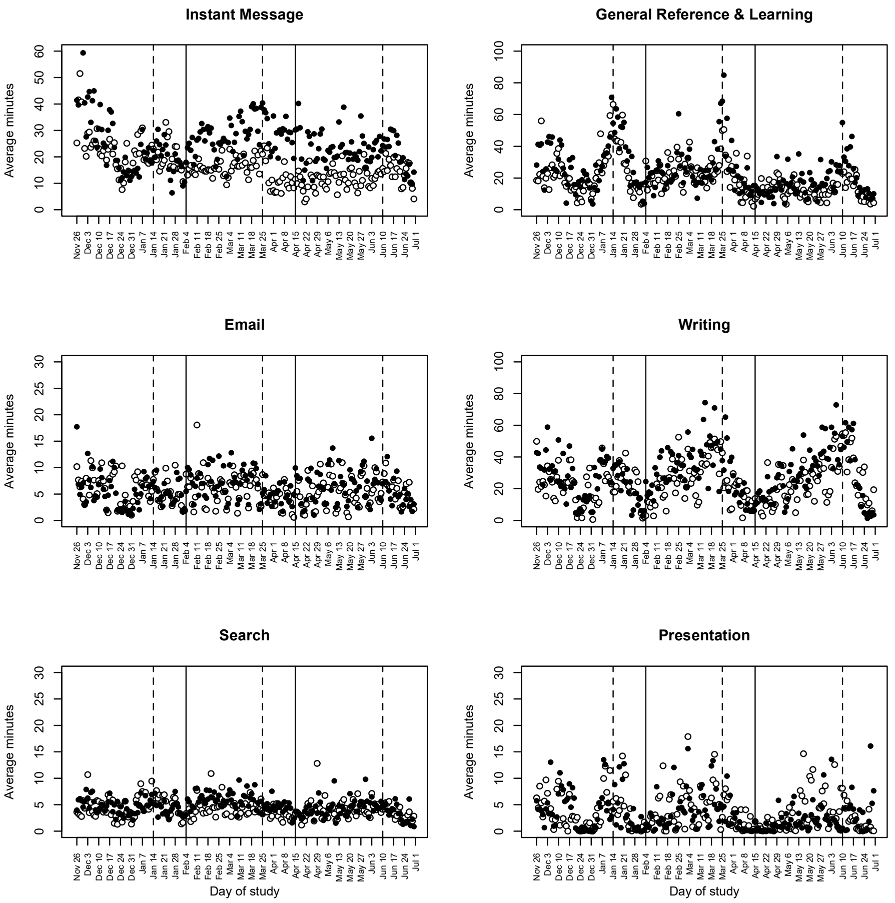

black = treatment, white = control group
solid vertical lines: start of a new teaching block
dashed vertical lines: start of the exam period

Figure 2.2: Tracked digital activities over time (users with Android devices)

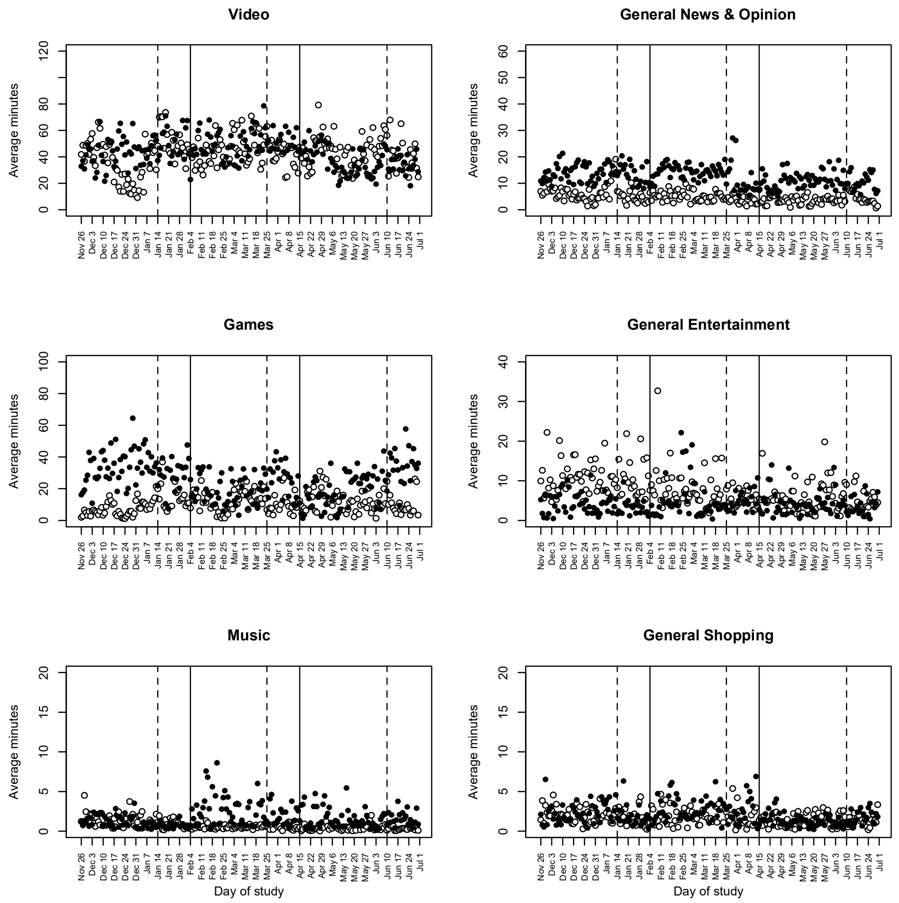

black = treatment, white = control group
solid vertical lines: start of a new teaching block
dashed vertical lines: start of the exam period

Figure 3: Differences in subjective well-being measures

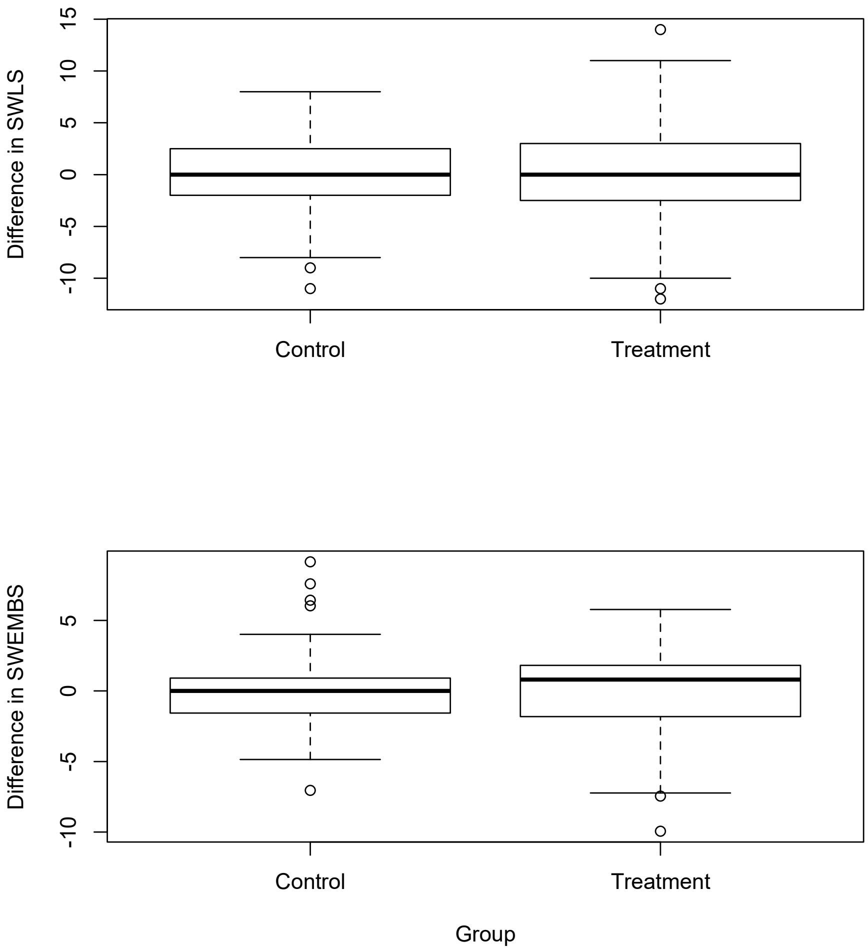

Figure 4: Differences in academic performance

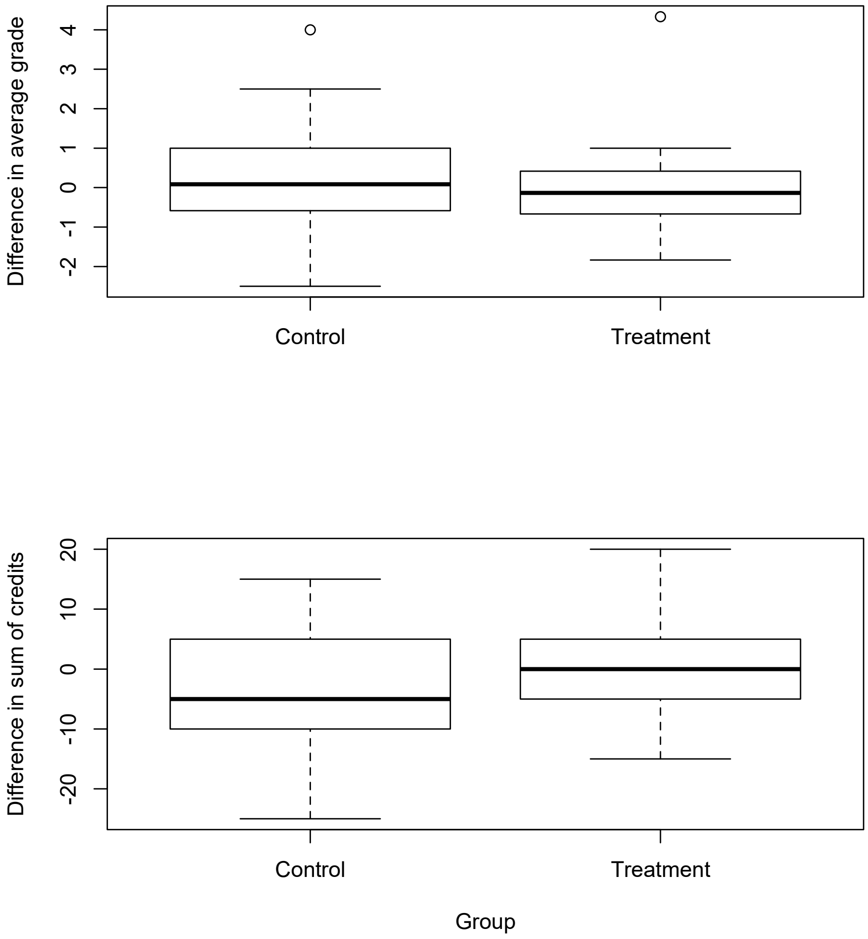

Supporting Information

Figure S1: All digital activities and social media usage over time (all users)

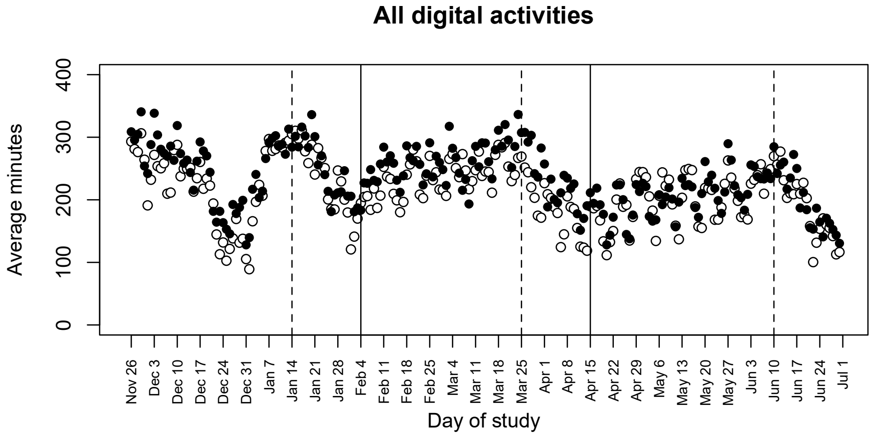

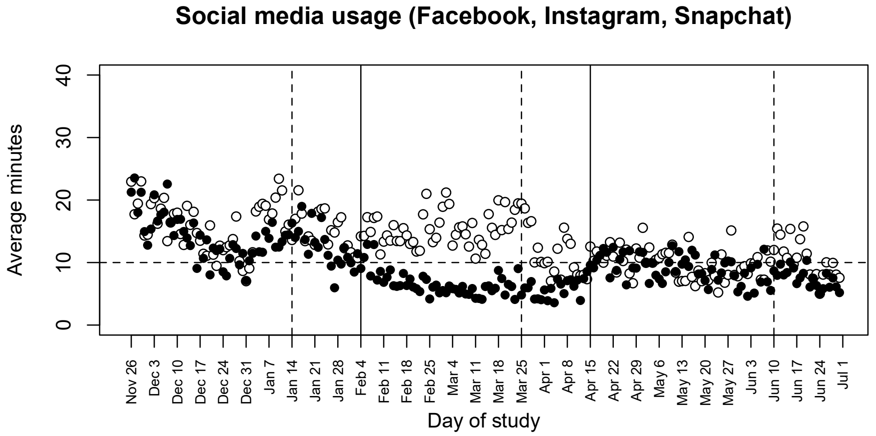

black = treatment, white = control group
solid vertical lines: start of a new teaching block
dashed vertical lines: start of the exam period

Figure S2.1: Tracked digital activities over time (all users)

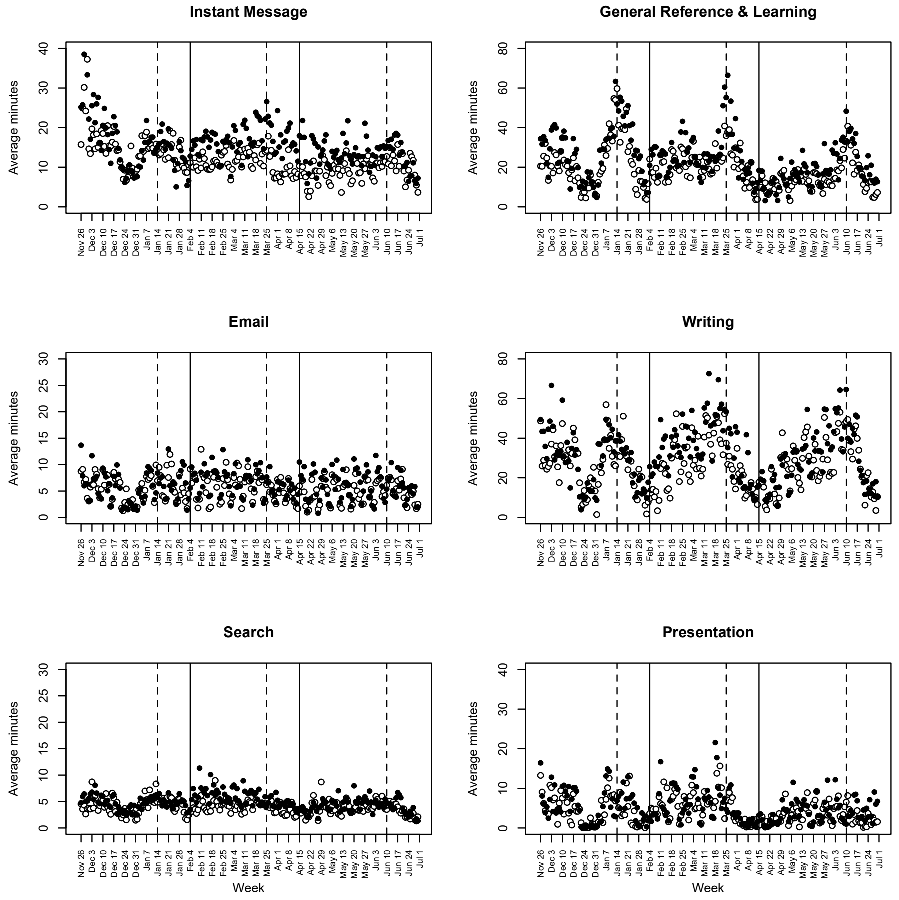

black = treatment, white = control group
solid vertical lines: start of a new teaching block
dashed vertical lines: start of the exam period

Figure S2.2: Tracked digital activities over time (all users)

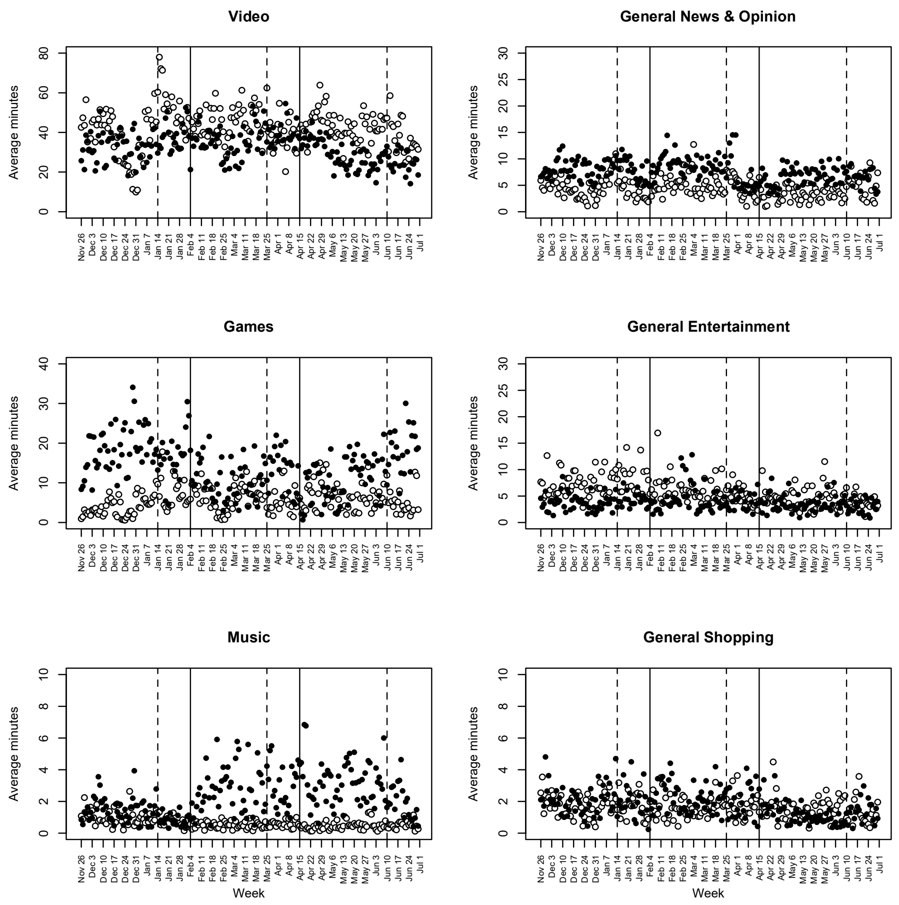

black = treatment, white = control group
solid vertical lines: start of a new teaching block
dashed vertical lines: start of the exam period

Table S1: Significance of differences between treatment and control group in block 2 vs. block 1 (difference-in-differences)

Category	p-value all users	p-value Android users
Social media	**<0.001**	**0.013**
General Reference & Learning	0.811	0.491
Instant Message	0.123	**0.008**
Browsers	0.628	0.257
Video	0.513	0.715
Writing	0.532	0.304
Search	0.745	0.494
Email	0.352	0.104
News & Opinion	0.864	0.543
General Entertainment	0.679	0.506
Games	0.411	0.662
Presentation	0.857	0.803
General Shopping	0.833	0.611
Music	0.099	**0.027**

All variables measured on a log scale.

Table S2: Summary statistics for well-being measures

Measure	Full sample		Treatment group		Control group	
	Mean	SD	Mean	SD	Mean	SD
SWLS survey 1	25.0	5.5	24.3	5.9	25.7	5.1
SWLS survey 2	25.0	5.4	24.4	5.4	25.6	5.3
SWLS survey 3	25.1	5.3	24.4	5.3	25.7	5.3
SWEMWBS survey 1	22.8	3.0	23.0	2.8	22.5	3.1
SWEMWBS survey 2	22.5	2.7	22.4	2.6	22.5	2.9
SWEMWBS survey 3	22.4	3.3	22.4	3.6	22.5	3.1

Table S3: Regression of satisfaction with life and mental well-being measures (standardized coefficients in parentheses)

	Satisfaction with life (SWLS)				Mental well-being (SWEMWBS)			
	All users		Android		All users		Android	
	Estimate	p-value	Estimate	p-value	Estimate	p-value	Estimate	p-value
(Intercept)	**29.028**	**<0.001**	**36.967**	**<0.001**	**24.409**	**<0.001**	**30.497**	**<0.001**
Treatment group	-1.205 (-0.113)	0.221	-1.350 (-0.117)	0.380	-0.233 (-0.038)	0.676	-0.063 (-0.009)	0.942
Block 2	0.207 (0.019)	0.831	-0.552 (-0.048)	0.720	0.025 (0.004)	0.963	-0.342 (-0.050)	0.691
(Treatment group*Block2)	-0.253 (-0.020)	0.855	1.285 (0.097)	0.552	-0.126 (-0.018)	0.872	0.505 (0.065)	0.676
Gender (female)	-1.021 (-0.096)	0.143	-0.818 (-0.070)	0.455	**-0.991 (-0.162)**	**0.013**	-0.768 (-0.112)	0.210
Age in years	-0.161 (-0.096)	0.221	-0.494 (-0.217)	0.068	-0.057 (-0.059)	0.446	**-0.338 (-0.252)**	**0.026**
Years at the university	0.166 (0.048)	0.542	0.107 (0.028)	0.798	-0.139 (-0.069)	0.370	-0.089 (-0.040)	0.705
Working next to studies	0.280 (0.026)	0.692	-0.036 (-0.003)	0.975	0.724 (0.117)	0.072	1.020 (0.150)	0.117
R-squared	0.032		0.051		0.055		0.142	

Thank you for making the extensive changes to your paper in response to the reviewers' and my suggestions. The paper is much stronger but I have a few more suggestions before moving on to making a decision on publication.

Thank you for your helpful comments. We have incorporated a number of changes following your suggestions and believe we have addressed all your remaining concerns. Responses to your comments are listed below in blue.

First, a very real concern is the statistical power of your study, and I don't think your description of that problem on page 27 is very clear or helpful. First of all, we don't usually consider power from the point of view of the maximum difference we cannot detect. It would be easier to understand this if you talked about the minimum difference you can detect.

Thank you. We have reformulated this section and other sections of the paper to refer to the minimum detectable difference. In addition, we now clarify the power calculation for our study in the Sample section.

Second, I am puzzled as to why you are talking about increases in life satisfaction, when the concern is for decreases.

Thank you for pointing this out, we have reworded this section and we do not refer to increases in life satisfaction anymore.

Also, should you not be citing the differences you observed and discussing whether you could have found those to be significant with more power? That is what seems to be the issue.

Yes, we agree. We made the following edits based on your comment: First, we only mention how we calculate power for our study in the Sample section and drop our earlier discussion on maximum difference that we cannot detect (see also above response). Second, we now directly discuss what we observed and what we could have been significant with more power in the regressions subsection of the results section. We are open to making further edits if you think our discussion is not clear.

Given that you found a slight increase among the Android group, it might make sense to look at both groups separately in considering the power question.

Thank you for this comment. While we agree with it, we have not performed this analysis as an analysis for each of the groups would reduce the sample size and lower the power further. We added a footnote to propose this as a direction for future research.

I would also recommend discussing power in the method section with how large a difference you will be able to detect given the sample size you have. Then in the Results, you could comment on whether your differences are large enough to have rejected the null hypothesis with more power.

Thank you. As mentioned previously, we now directly address power in the Methodology (Sample) and Results sections in detail. We explain power calculation in the Sample section and comment on whether the differences we find are large enough to have rejected the null hypothesis with more power in the Results section.

I am also puzzled as to why you show the regressions for grades but not for wellbeing and life satisfaction in the Results. It seems to me that the mental health concerns are the ones that motivated this to begin with, so those findings should receive priority.

Thank you. We have now added regressions for well-being to the main text and now present them before the regressions of academic grades.

Finally, I think your findings could use more discussion than you devote to them. It seems that your intervention to reduce some forms of social media use served to increase other forms of interaction within one's social network. So, what your findings seem to say is that social media may primarily serve as a way to connect with others and that arbitrarily defining those media as you have merely caused your students to migrate to other platforms that serve the same purpose. In other words, what your study shows is that with all of the ways that people can interact online, it is difficult to isolate some as more responsible for adverse effects. You would have to restrict the ability to use online platforms to communicate with others as a way to isolate that effect and I doubt that you could recruit a sample to do that for any length of time.

Thank you for nudging us to add more discussion. We now discuss how our definition of social media is just one way and future research can look at a broader set of apps

including instant messaging apps. We do not want to make the paper wordier, but we are happy to further extend the discussion if you think we are still missing something.

One other possible way that some social media may be harmful is by pushing advertising to users that they may not want exposure to and doing this to maintain continued engagement. This might be a mechanism for internet addiction that could be studied. In any case, I think a deeper discussion of these issues would be helpful for readers. Your point about the policy implications also follows from this, since many online media serve the same purpose and just differ in how they afford that opportunity.

We add this point to the discussion section.

I also think you have more findings to discuss, such as correlations between mental health and various uses of social and other media, such as in Table 4. Perhaps it is the video features of online media that are the source of adverse effects on mental health? And perhaps the sites push certain videos in order to maintain engagement?

Unfortunately due to the limitations of our data, we cannot observe what subjects do once they are on a social media app and what type of videos they watch on video streaming platforms. However, we add this as an avenue for future research in the discussion section.

In the abstract, I would describe the finding by saying that “we do not find any significant impact of social media usage as it was defined in this study on well-being…” As part of your limitations section, I would also take note of the fact that other forms of social media were not excluded in your study and this will be an obvious point of criticism.

Thank you for this comment. We have changed the abstract and added a part in the limitations section.

I also think it would be useful to consider a paper that just came in Clinical Psych Science by Sewall et al. They also find that various uses of digital tech are not very strongly related to mental health in young adults

Thank you for pointing out this new reference. We now added it to our literature review.
